# Processivity and specificity of histone acetylation by the male-specific lethal complex

**DOI:** 10.1093/nar/gkae123

**Published:** 2024-02-26

**Authors:** Anna E Kiss, Anuroop V Venkatasubramani, Dilan Pathirana, Silke Krause, Aline Campos Sparr, Jan Hasenauer, Axel Imhof, Marisa Müller, Peter B Becker

**Affiliations:** Biomedical Center, Molecular Biology Division, Ludwig-Maximilians-University of Munich, Planegg-Martinsried, Germany; Biomedical Center, Molecular Biology Division, Ludwig-Maximilians-University of Munich, Planegg-Martinsried, Germany; Life and Medical Sciences (LIMES) Institute, Rheinische Friedrich-Wilhelms-Universität Bonn, Bonn, Germany; Biomedical Center, Molecular Biology Division, Ludwig-Maximilians-University of Munich, Planegg-Martinsried, Germany; Biomedical Center, Molecular Biology Division, Ludwig-Maximilians-University of Munich, Planegg-Martinsried, Germany; Life and Medical Sciences (LIMES) Institute, Rheinische Friedrich-Wilhelms-Universität Bonn, Bonn, Germany; Computational Health Center, Helmholtz Zentrum München Deutsches Forschungszentrum für Gesundheit und Umwelt (GmbH), Neuherberg, Germany; Biomedical Center, Molecular Biology Division, Ludwig-Maximilians-University of Munich, Planegg-Martinsried, Germany; Biomedical Center, Molecular Biology Division, Ludwig-Maximilians-University of Munich, Planegg-Martinsried, Germany; Biomedical Center, Molecular Biology Division, Ludwig-Maximilians-University of Munich, Planegg-Martinsried, Germany

## Abstract

Acetylation of lysine 16 of histone H4 (H4K16ac) stands out among the histone modifications, because it decompacts the chromatin fiber. The metazoan acetyltransferase MOF (KAT8) regulates transcription through H4K16 acetylation. Antibody-based studies had yielded inconclusive results about the selectivity of MOF to acetylate the H4 N-terminus. We used targeted mass spectrometry to examine the activity of MOF in the male-specific lethal core (4-MSL) complex on nucleosome array substrates. This complex is part of the Dosage Compensation Complex (DCC) that activates X-chromosomal genes in male *Drosophila*. During short reaction times, MOF acetylated H4K16 efficiently and with excellent selectivity. Upon longer incubation, the enzyme progressively acetylated lysines 12, 8 and 5, leading to a mixture of oligo-acetylated H4. Mathematical modeling suggests that MOF recognizes and acetylates H4K16 with high selectivity, but remains substrate-bound and continues to acetylate more N-terminal H4 lysines in a processive manner. The 4-MSL complex lacks non-coding *roX* RNA, a critical component of the DCC. Remarkably, addition of RNA to the reaction non-specifically suppressed H4 oligo-acetylation in favor of specific H4K16 acetylation. Because RNA destabilizes the MSL-nucleosome interaction *in vitro* we speculate that RNA accelerates enzyme-substrate turn-over *in vivo*, thus limiting the processivity of MOF, thereby increasing specific H4K16 acetylation.

## Graphical Abstract

**Graphical Abstract:**
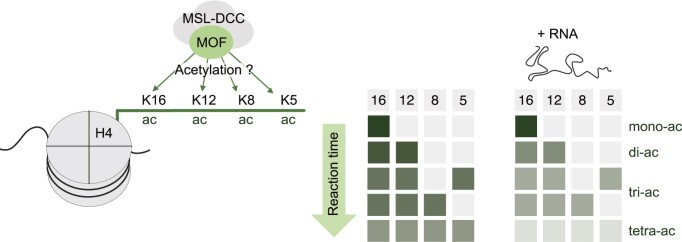

## Introduction

The expression of genes depends on their chromatin organization. Post-translational modification of histones constitutes a means through which chromatin structure can be modulated to regulate transcription. One well-explored type of modification is the acetylation of the N-terminal ‘tail’ domains of histones H3 and H4, known primarily for its role in activating transcription.

The underlying mechanisms can be manifold (1). Acetylation neutralizes the positive charge of lysines, which weakens histone-DNA interactions leading to a general decompaction of chromatin. Acetylated lysines can also be bound by dedicated ‘reader’ domains, of which bromodomains are best known. Chromatin modifiers and transcription regulators containing bromodomains may thus be targeted to sites of specific lysine acetylation to exert their functions (2). In contrast to acetylation, the methylation of lysines in histone N-termini in some cases constitutes repressive chromatin. Thus, acetylation of lysines often opposes the placement of repressive methyl-marks.

Depending on the underlying mechanism, the acetylation of lysines at specific positions in the histone N-termini may have distinct effects. One prominent example for such specificity involves acetylation of lysine 16 of histone H4 (H4K16ac). K16 is part of the ‘basic patch’ of histone H4, consisting of amino acids 16–20, which can interact with a corresponding ‘acidic patch’ on the H2A-H2B dimer on close-by nucleosomes, thus stabilizing the folding of the nucleosome array (3–5). Acetylation of H4K16 disrupts this interaction leading to unfolding of the nucleosome fiber (6,7).

Other lysines on the H4 N-terminus (K5, K8 and K12) have different functions. Newly synthesized H4 is acetylated at K5 and K12 and deacetylated after chromatin assembly (8–10). The acetylation of H4K12 is also often found at promoters, where it may be read out by bromodomain effectors (11).

The site-specific functions of lysine acetylation are mirrored by the substrate selectivity of the enzymes that place them. Different lysine acetyltransferases (KATs) acetylate individual lysines with varying degrees of selectivity and redundancy in histones, but also many non-histone substrates (1).

KATs are generally conserved across species and can be categorized into five enzyme families based on their histone acetyltransferase (HAT) domains (12,13). The MYST family members share a conserved HAT domain named ‘MYST’, after its founding members MOZ, Ybf2/Sas3, Sas2 and Tip60 (13). MYST-type KATs usually reside in large, multiprotein complexes (14,15). The additional complex subunits often contain domains that recognize histone modifications, transcription factors or other chromatin constituents and may therefore target the KAT to specific sites in chromatin or regulate its enzymatic activity.

In this study, we focused on the MYST-type acetyltransferase MOF (males absent on the first, KAT8), which is the effector enzyme of two transcription regulator complexes in *Drosophila melanogaster*. As part of the male-specific lethal dosage compensation complex (MSL-DCC, or DCC for short), the enzyme is targeted to transcribed genes on the X chromosome, where it acetylates H4K16 to boost transcription (16–18). In the context of the non-specific lethal (NSL) complex, MOF has been reported to acetylate different combinations of histone H4 lysines 5, 8, 12 and 16 at promoters in different cells, illustrating the potential of complex subunits to modulate substrate specificity (19–22).

Defining the substrate selectivity of KATs *in vivo* poses several challenges (1). The intrinsic substrate selectivity of enzymes may be modulated by its molecular environment, be it the complex assembly or the chromatin context. Redundant activities of different enzymes, indirect effects and the action of lysine deacetylases (KDACs) may occlude the consequences of loss-of-function manipulation. Furthermore, the specificity of the antibodies that are commonly used to detect distinct acetylated lysines in histones may be compromised due to low-level off-target effects, interference of modifications next to acetylated lysines, and the tendency of Kac-antibodies to react with relaxed selectivity with oligo-acetylated histone tails (23–26). A more reliable strategy involves the detection of modified histone peptides by mass spectrometry, which unequivocally detects individual modifications and defined combinations of modifications and has a much better dynamic range than traditional Western blotting (27–31).

In a complementary approach one may determine the intrinsic substrate selectivity of defined recombinant enzymes and KAT complexes in biochemical assays (32). We recently reconstituted a recombinant MOF-containing core DCC [here termed ‘4-MSL’, since it contains the subunits MOF, MSL1, MSL2 and MSL3 (33)] and now report on a study of its substrate selectivity on recombinant nucleosome arrays. Pilot experiments using traditional Western blotting suggested that, in addition to the expected H4K16 acetylation, H4K12 was modified. Since this finding disagrees with the prevailing view that the DCC selectively acetylates H4K16, we applied targeted mass spectrometry to analyze acetylation of the H4 N-terminus. We compared the acetylation reaction of the 4-MSL complex with another MYST enzyme, dTip60 (KAT5), in the context of a trimeric complex, akin to the yeast piccolo NuA4 complex, which is thought to have a more relaxed lysine selectivity on the H4 N-terminus (34–36).

We found that the 4-MSL complex acetylated H4K16 with excellent selectivity, while the dTIP60^piccolo^ complex preferentially modified H4K12. To our surprise, we also observed that the 4-MSL complex progressively acetylated K12, K8 and K5 during longer incubations, leading to a complex mixture of oligo-acetylated H4. Mathematical modeling suggests that MOF initially acetylates H4K16 with high selectivity, but remains bound to the substrate and processively acetylates other N-terminal H4 lysines, contrasting the supposed physiological K16ac selectivity.

Because the MSL-DCC *in vivo* contains long non-coding *roX* RNA (16,37–39), we explored whether RNA affected the specificity of the reaction. We observed that the addition of RNA to the HAT reaction improves the specificity of the reaction and suppresses the generation of oligo-acetylated forms, possibly by reducing the dwell time of the enzyme on the nucleosome substrate.

The comparison of MOF and dTip60 activities in the context of recombinant HAT complexes revealed significant differences in activity and specificity. Our study describes a powerful experimental approach and conceptual framework for the analysis of physiologically relevant components that modulate intrinsic activities.

## Materials and methods

### Cloning, protein expression and purification

*Drosophila melanogaster* histones (wild-type and H4K16R mutant, respectively) were expressed and purified as described (40). The histone H4K16R mutation was introduced into codon-optimized *Drosophila melanogaster* H4 expression plasmid in a pET3c vector (40) using the QuikChange site directed mutagenesis kit (Biolabs). Primers are given in the Supplementary Table.

Cloning of *Drosophila* 4-MSL complex and MLE were described earlier (33,41).

The 3-subunit dTIP60^piccolo^ complex was cloned and expressed using the biGBac technology (42). The cDNAs of full-length *Drosophila melanogaster* E(Pc), Ing3 and dTip60 fused to an N-terminal TwinStrep tag were combined in one pBIG1 vector. Primers are given in the Supplementary Table. The bacmid was transfected into *Spodoptera frugiperda* 21 (Sf21) cells to produce one dTIP60^piccolo^-expressing baculovirus.

*Drosophila* 4-MSL complex, MLE and the dTIP60^piccolo^ complex were expressed in Sf21 cells infected with 1/1000 (v/v) baculovirus for 72 h at 26°C. Cells were collected by centrifugation, washed once with PBS buffer, flash frozen in liquid nitrogen and stored at –70°C.

The 4-MSL complex was purified from isolated nuclei of 5 × 10^8^ baculovirus-infected Sf21 cells as described (33) with minor modifications (all steps at 4°C). Nuclei were solubilized in 5 ml buffer NE-B (20 mM HEPES pH 7.0, 400 mM KCl, 2 mM EDTA, 1 mM EGTA, 0.5 mM PMSF, 1 mM DTT) and incubated for 15 min with head-over-end rotation. The lysate was diluted with 1 volume of PBS (140 mM NaCl, 2.7 mM KCl, 10 mM Na_2_HPO_4_, 1.8 mM KH_2_PO_4_) supplemented with 1 mM DTT, 50 μM ZnSO_4_ and 0.2 mM PMSF, and centrifuged for 10 min at 17 000 g. The supernatant was incubated on 1.5 ml 50% suspension FLAG-affinity beads (Anti-DYKDDDDK G1 Affinity Resin, Genscript Corporation) for 1 h with head-over-end rotation in presence of 400 μg RNase A (Sigma-Aldrich). Beads were washed three times (PBS with 1 mM DTT, 50 μM ZnSO_4_; PBS + 200 mM KCl; PBS only). The protein complex was eluted in three subsequent steps of 1 bead volume elution buffer each (PBS, 0.5 mg/ml FLAG peptide (Sigma-Aldrich)) for 30 min. Pooled elution fractions were supplemented with 10% (v/v) glycerol, flash-frozen in liquid nitrogen and stored at –70°C. Protein concentration was determined by SDS-PAGE and Coomassie staining using ImageLab (Bio-Rad, version 6.0) and BSA standard kit (Thermo Fisher Scientific) as reference.

FLAG-tagged MLE was expressed in Sf21 insect cells and purified by FLAG-affinity chromatography as described in (41).

The dTIP60^piccolo^ complex was purified from 2.5 × 10^8^ baculovirus-infected Sf21 cells (all steps at 4°C). The cell pellet was resuspended in 25 ml lysis buffer (25 mM HEPES pH 7.5, 300 mM KCl, 5% glycerol, 0.05% NP-40, 3 mM MgCl_2_, EDTA-free protease inhibitor (Roche) and supplemented with 200 μg of RNase A (Sigma-Aldrich) and 4 μl of benzonase (Merck Millipore). The suspension was incubated for 15 min on ice. Following sonication (Branson sonifier, 60 s, 20% amplitude), cell debris was removed by centrifugation at 30 000 g for 30 min. The supernatant was transferred to a fresh tube and filtered twice through a Millex HPF filter 0.45 μm pore size. The filtered cell lysate was loaded onto a 2 ml StrepTrap Streptactin column (Cytiva), equilibrated with wash buffer 200 (25 mM HEPES pH 7.5, 5% glycerol, 3 mM MgCl_2_, 200 mM KCl). The column was washed with 10 CV wash buffer 300 (300 mM KCl). Proteins were eluted in 10 CV biotin elution buffer (50 mM biotin pH 8.0, 25 mM HEPES pH 7.5, 150 mM NaCl, 10% glycerol, 3 mM MgCl_2_, 1 mM EDTA) and analyzed by SDS-PAGE and spectrophotometry at 280 nm. dTip60-containing fractions were pooled, concentrated using a 50000 Da cut-off concentrator (Amicon), flash-frozen in liquid nitrogen and stored at –70°C. Protein concentration was determined by SDS-PAGE and Coomassie staining using ImageLab (Bio-Rad, version 6.0) and BSA standards (Thermo Fisher Scientific) as reference.

### Cell lines

For amplification of recombinant baculoviruses and expression of recombinant proteins, Sf21 cells (Gibco) were cultured at 26°C in Sf-900 II medium (Gibco) supplemented with 5% FCS and gentamycin. *Drosophila melanogaster* Kc cells (Drosophila Genomics Resource Center) were cultured in Schneider's Drosophila medium (Gibco) supplemented with 10% FCS and penicillin-streptomycin at 26°C.

### Histone octamer assembly

Histone octamers (wild-type and H4K16R, respectively) were assembled as described in Luger et al. 1997 (43). Briefly, individual lyophilized histones were resuspended in unfolding buffer (7 M guanidinium-Cl, 20 mM Tris pH 7.5, 10 mM DTT), mixed in a molar ratio of 1.4:1.4:1:1 (H2A:H2B:H3:H4) and dialyzed for 16 h against 2 l of refolding buffer (2 M NaCl, 10 mM Tris pH 7.5, 1 mM EDTA, 1 mM DTT). After two additional dialysis steps of 1 h each against 1 l of refolding buffer at 4°C, samples were centrifuged for 30 min at 30 000 g at 4°C. The supernatant was loaded onto a Superdex 200 HiLoad chromatography column (Cytiva) equilibrated in refolding buffer, which was also used as running buffer. Octamer-containing fractions were identified by UV absorption at 280, 254 and 214 nm wavelength and analyzed by SDS-PAGE. Concentration was measured by UV absorption at 280 nm in a Nanodrop device. Fractions were pooled and concentrated to >1 μg/μl, frozen in liquid N_2_ and stored at –70°C.

### Assembly of nucleosome arrays by salt gradient dialysis

Nucleosome arrays were assembled by salt gradient dialysis (44–46) on a pUC18 plasmid comprising 25 repeats of a 197 bp long Widom-601 nucleosome positioning sequence (47–49). The plasmid DNA was mixed with octamer in a 1.1:1 mass ratio in a high salt buffer (10  mM Tris–HCl pH 7.6, 2  M NaCl, 1  mM EDTA, 0.05% IGEPAL CA630, 14.3  mM β-mercaptoethanol). The salt was gradually reduced by dialysis overnight at 30°C for 15–18 h to low salt buffer (10  mM Tris–HCl pH 7.6, 50  mM NaCl, 1  mM EDTA, 0.05% IGEPAL CA630, 1.4  mM β-mercaptoethanol). The dialysis was continued on the next day for 1 h against fresh low salt buffer. Nucleosome array concentration was determined based on DNA absorption at 260 nm wavelength. Nucleosome arrays were stored at 4°C for up to 12 weeks.

Nucleosome array assembly quality was evaluated by micrococcal nuclease (MNase) digest as described (45). 500 ng nucleosome arrays were mixed with 3 μl of 1.5 U/μl units MNase (Sigma-Aldrich) in MNase buffer (20 mM HEPES pH 7.5, 50 mM NaCl, 3 mM MgCl_2_, 2.5 mM DTT, 0.5 mM EGTA, 1.5 mM CaCl_2_) and incubated for 30 s, 60 s or 5 min at 30°C. The reaction was stopped by addition of 10 mM EDTA and 2% (w/v) SDS. The sample was treated with 2.5 μl Proteinase K (10 mg/ml, Bioline) for 30 min at 37°C. DNA was precipitated with 325 μl 100% ethanol and 65 mM NaCl for 20 min at –20°C, followed by centrifugation at 21 000 g at 4°C for 30 min and washed once with 400 μl of 70% ethanol. The pellet was air-dried and resuspended in 15 μl 10 mM Tris–HCl pH 8.0 and 3 μl of Orange G loading dye (NEB). The digestion degree was analyzed on a 1.5% (w/v) agarose gel in 1x TAE buffer, stained with Midori green (Nippon Genetics).

### Mononucleosome assembly by salt gradient dialysis

Mononucleosomes were assembled with *Drosophila* histone octamers and the 147 bp Widom 601 DNA (50) with 80 bp extranucleosomal DNA in the 0N80 orientation. DNA was amplified by PCR from a plasmid template (51) and fluorescently labeled at the extranucleosomal end using a 6-carboxyfluorescein (6-FAM) labeled reverse primer. The plasmid template was eliminated by precipitation with 0.5 vol of a 30% PEG 8000/30 mM MgCl_2_ mixture and centrifugation (17 000 g, 20 min, 25°C). Labeled DNA was precipitated from the supernatant by adding 1 vol isopropanol and incubation at 25°C for 30 min. Following centrifugation (20 000 g, 30 min, 25°C), the DNA pellet was washed with 70% ethanol and air-dried. DNA was resuspended in TE buffer (10 mM Tris–HCl pH 8.0, 0.1 mM EDTA) and photometrically quantified. To assemble mononucleosomes, a 1.1-fold molar excess of 6-FAM-labeled DNA was mixed with histone octamer at 2 M NaCl. The salt concentration was gradually decreased to 50 mM by dialysis over 18 h at 4°C. Mononucleosome concentration was determined based on DNA absorption at 260 nm wavelength.

### RNA preparation

*In vitro* transcription of *roX2* and *GFP* RNA was performed using the MegaScript T7 RNA polymerase kit (Ambion) or T7 polymerase (NEB with Ambion buffers and nucleotides), following the manufacturer's instructions. The DNA templates were prepared by restriction enzyme digestion with XbaI (NEB) in the case of *roX2* constructs as described (41). GFP templates were amplified by PCR (for primers, see Supplementary Table) from an pHSP70-MLE-GFP plasmid (52). After transcription (4 h, 37°C) and DNase I treatment (30 min, 37°C), RNA products were purified by denaturing PAGE (8 M urea, 5% acrylamide, 1x TBE), phenol:chloroform:isoamylalcohol (Roth) extraction and ethanol precipitation. RNA was dissolved in 50 μl of nuclease-free water (Ambion) and stored at –70°C. Total RNA from female *Drosophila* Kc cells for EMSA was extracted using the RNeasy Mini Kit (Qiagen).

### Histone acetyltransferase (HAT) assay

0.8 μg 25× 'Widom-601' nucleosome arrays and protein complexes were incubated in HAT buffer (50 mM HEPES pH 7.9, 3 mM MgCl_2_, 50 mM KCl and 10 μM acetyl-CoA (Sigma-Aldrich)) at 26°C. Protein concentration (12.5–175 nM) and incubation times (2–180 min) varied between the different sets of experiments and are provided in the respective figures. HAT reactions with the 4-MSL complex and RNA included 1 mM ATP (Sigma-Aldrich), 1 U RNasin RNase inhibitor (Promega) and MLE at a 1:1 molar ratio to the 4-MSL complex. RNAs were titrated (25–200 nM) or applied in 2-fold excess as mass ratios. HAT reactions with dTIP60^piccolo^ were performed with 50 nM protein complex and incubation times from 5 to 60 min. When indicated, 100 nM *GFP* RNA (470 bases length) and 0.2 μg RNase A were added. The reaction was stopped by incubation at 95°C for 5 min. Histone acetylation was analyzed by Western blot or by mass spectrometry (see below). The denatured HAT reaction samples were supplemented with SDS-PAGE loading buffer and boiled before loading onto a 15% (v/v) acrylamide Bis-Tris-MES gel in 1× MES buffer (50 mM MES, 50 mM Tris, 1 mM EDTA, 0.1% SDS, 5 mM sodium metabisulfite). Proteins were separated at 140 V constant and transferred (1 h, 400 mA, 4°C) onto a nitrocellulose membrane in transfer buffer (25 mM Tris, 192 mM glycine). Membranes were blocked in 3% (w/v) BSA in TBS for 1 h at RT and probed overnight at 4°C with antibodies specific for histone H3 (anti-H3, mouse, Abcam ab10799) and histone H4 acetylation (anti-H4K16ac, rabbit, Millipore 07-329; anti-H4K12ac, rabbit, Millipore 07-595), respectively. Membranes were washed three times with TBS-T and incubated with species-specific secondary antibodies (LI-COR). Images were acquired using the LI-COR imaging system.

### Electrophoretic mobility shift assay (EMSA)

Interaction of the 4-MSL complex and 6-FAM labeled 0N80 mononucleosomes in absence or presence of RNA and MLE, respectively, was monitored in electrophoretic mobility shift assays (EMSAs). In a 20 μl reaction, 10 nM mononucleosomes were incubated with 50 nM of 4-MSL complex in EMSA buffer (25 mM HEPES pH 7.9, 60 mM KCl, 7% glycerol, 0.5 mM DTT) for 20 min on ice. Where indicated, 50 nM MLE or the following RNA variants were added to the reaction: *roX2* (552 bases; 25–100 nM); *GFP* (627/550/470/391/283/182/76 bases; 25–100 nM); *Drosophila* Kc total RNA, yeast tRNA (Sigma-Aldrich-Aldrich), poly(dA) (Roche): all 200 ng, equivalent mass to 50 nM *roX2*. Samples were analyzed at 4°C by native PAGE on a 3–12% acrylamide Bis–Tris gel (ThermoFisher Scientific) in 0.5× Native PAGE running buffer (ThermoFisher Scientific) and visualized using the Cy2 laser of the Typhoon imaging system (GE Healthcare).

### Sample preparation for mass spectrometry

Samples for mass spectrometry were prepared as described (27). Briefly, heat-denatured HAT assay samples were chemically acetylated using 25% (v/v) fresh acetic anhydride-D6 (Sigma-Aldrich) for 1 min at RT. The mass difference of three Daltons allows distinguishing chemical and enzymatic acetylation. Light and heavy isotope-labeled acetyl groups have the same chemical properties allowing for reliable MS quantification (27). The pH was adjusted stepwise to pH 7.0 by addition of 1 M ammonium bicarbonate. D6-acetylation continued for 45 min at 37°C with 500 rpm agitation. Proteins were digested with 1 μg of trypsin for 16 h at 37°C with 500 rpm agitation. C18 STAGE tips were prepared for desalting as described (53). They were washed with 60 μl each of (i) 100% acetonitrile, (ii) 0.1% (v/v) trifluoroacetic acid, 80% (v/v) acetonitrile in MS-grade water and (iii) 0.1% (v/v) trifluoroacetic acid in MS-grade water before samples were loaded to the C18 tips. Liquid was passed through by centrifugation at 300–400 g for 3 min at RT. Trypsin digested samples were loaded to the C18 STAGE tips. The flow through was loaded once again. Bound peptides were washed three times with 0.1% (v/v) trifluoroacetic acid in MS-grade water. Peptides were eluted in three steps with 0.25% (v/v) trifluoroacetic acid, 80% (v/v) acetonitrile in MS-grade water. The samples were dried under vacuum in a speed vac for 1 h at 45°C. Samples were resuspended in 17 μl MS buffer (0.1% (v/v) trifluoroacetic acid in MS-grade water), sonicated for 5 min and stored until measured at –20°C.

### Mass spectrometry analysis of histone modifications

Samples were separated in an Ultimate 3000 RSLCnano system (Thermo) using a 25-cm Aurora column (Ionopticks) with a 50-min gradient from 2 to 37% acetonitrile in 0.1% formic acid. The effluent from the HPLC was directly electrosprayed into a Qexactive HF (Thermo). The MS instrument was programmed to target several ions as described previously (27) except for the MS3 fragmentation. For the analysis of the H4K16R mutant, the m/z values of the corresponding peptide ions [M + H2]2 + were adjusted accordingly (H4G4R17 monoacetylated adjusted from 724.9428 to H4G4R16 637.8759, diacetylated from 723.4329 to 636.3665 and triacetylated from 721.9221 to 634.8571). Survey full scan MS spectra (from m/z 270–730) were acquired with resolution *R* = 60 000 at *m*/*z* 400 (AGC target of 3 × 10^6^). Targeted ions were isolated with an isolation window of 0.7 *m*/*z* to a target value of 2 × 10^5^ and fragmented at 27% normalized collision energy. Typical mass spectrometric conditions were: spray voltage 1.5 kV; no sheath and auxiliary gas flow; heated capillary temperature, 250°C.

### Data analysis of MS data post-translational modification of histones

Raw data from mass spectrometry was analyzed using Skyline (54) v21.1. Peak integration was performed for H4 peptides for each of its corresponding modifications. Relative levels of each PTM were calculated from the obtained intensities using R (Supplementary Note S3), based on the formula given in (27). Formula to calculate the relative levels of K16R mutated H4 motifs are given in Supplementary Note S1. MS1 spectra of acetylated wild-type and H4K16R mutant peptides were generated using Xcalibur 4.4 using the dataset of replicate 3.

### Mathematical modeling

Mathematical modeling was performed to rank hypotheses by their ability to explain experimental data of time-dependent histone H4 acetylation by MOF on wild-type (Figure 2) and K16R mutant nucleosome arrays (Figure 4), respectively. The mathematical modeling was based on the framework described in Blasi *et al.* (55). All 16 acetylation states of the H4 tail (‘motifs’) were included in each model. Several hypotheses for the reaction mechanism were tested (see Figure 3 and Supplementary Note S2 for further details). Each of these hypotheses was modeled with ordinary differential equations (ODEs). The ODEs describe the instantaneous change in the acetylation process, and when solved provide time course simulations of motif concentrations. The ODEs are fully specified in the Systems Biology Markup Language (SBML) (56) and are provided in Supplementary Note S2. A schematic description of the kinetics in each model of ODEs is provided in Figure 3A.

Each model was calibrated using maximum likelihood estimation. The framework is described in full in Supplementary Note S2, and the full specification of each parameter estimation problem in the Parameter Estimation tables (PEtab) format (57) is provided at Zenodo (DOI: 10.5281/zenodo.10221453). The Akaike Information Criterion (AIC) was used to compare the models. A difference in the AIC values of 10 was considered sufficient to reject the model with the higher AIC value (58). As the mass spectrometry directly measures the relative motif abundance, the observation model was a direct mapping between motifs in the model and measured motifs. The parameter uncertainties were quantified using sampling of the Bayesian posterior distribution (assuming bounded uniform priors for all parameters). The sample in the converged chain were then simulated, and 99% credibility intervals were generated for model predictions.

The AMICI (59) interface to the CVODES solver in the SUNDIALS suite (60) was used to solve the ODEs and compute sensitivity-based gradients. The Python Parameter EStimation TOolbox (pyPESTO) (61) was used for parameter estimation, with the Fides optimizer (62) and adaptive parallel tempering (61).

## Results

### MOF in the 4-MSL complex acetylates H4K16 and H4K12 on nucleosome arrays *in vitro*

Recombinant MOF acetylates isolated histone H4, but is unable to use nucleosomes as substrate. The enzyme gains the ability to acetylate nucleosomal H4 in complex with MSL1 and MSL3 (63). We assayed MOF in the context of the 4-MSL complex, which contains equimolar amounts of MOF, MSL1, MSL2 and MSL3. Each HAT assay involved three biological replicates, which in this biochemical assay corresponds to three different enzyme preparations (Supplementary Figure S1A). The HAT activity was assayed on nucleosome arrays assembled by salt gradient dialysis from recombinant *Drosophila* histones on a plasmid containing 25 ‘Widom-601′ nucleosome positioning sequences (47–49). Several independent substrate preparations were used, which were quality-controlled by partial Micrococcal Nuclease (MNase) cleavage (Supplementary Figure S1B).

**Figure 1.**
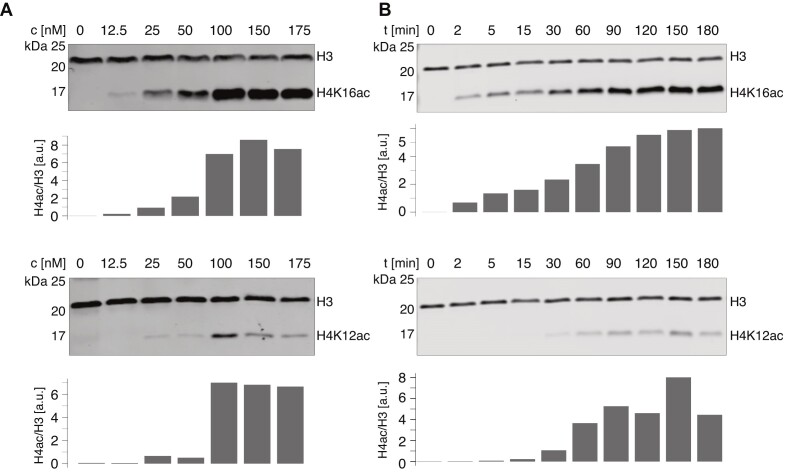
The 4-MSL complex acetylates H4K16 and H4K12 on a nucleosome array substrate *in vitro*. (**A**) Representative histone acetylation assay on nucleosome array substrate with purified 4-MSL complex at indicated concentrations (*c*). Reaction time was 60 min. Acetylation of histone H4 was detected by Western blot using antibodies specific for H4K16ac and H4K12ac, respectively. Histone H3 served as loading control and was used to quantify relative H4 acetylation as represented in the bar plot as arbitrary units (a.u.). (**B**) Representative histone acetylation assay using 50 nM 4-MSL complex at reaction times (*t*) ranging from 2 to 180 min. In the ‘0′ lane reaction the 4-MSL was omitted. Detection and quantification of H4 acetylation as in (A).

In pilot experiments, H4K16 acetylation was detected by Western blotting using a specific antibody and the signal was normalized to the signal corresponding to unmodified histone H3, detected with a second antibody. To determine the linear range of the acetylation reaction, a range of substoichiometric concentrations (12.5–175 nM) of 4-MSL were assayed on 200 nM nucleosomes during a 60-min reaction (Figure 1A and Supplementary Figure S1D). No H4K16ac was detected in a control reaction lacking enzyme. The expected H4K16ac mark was faintly detected already at 12.5 nM 4-MSL with a steady signal increase, until at 150 nM enzyme saturation was reached. A concentration of 50 nM 4-MSL complex fell into the linear range of the assay and was chosen for all further experiments.

To assess the specificity of the reaction the membrane was probed with an antibody that detects H4K12ac. The H4K12ac signal was substantially weaker, to our surprise nevertheless detectable at a 4-MSL concentration of 25 nM and increased to saturation at 100 nM.

Next, we compared the acetylation efficiency on nucleosome arrays, which were assembled in different DNA:octamer stoichiometries. While over-assembled arrays (ratio 0.5) showed slightly reduced H4K16ac in Western blot, under-assembled arrays (ratio 2) resulted in an increase in H4K16ac, which likely reflects better accessibility for acetylation in lesser assembled arrays (Supplementary Figure S1C). Nucleosome arrays with an assembly degree of DNA:octamer of 1:0.9 were chosen for all experiments to avoid over-assembly.

To identify the linear range of MOF activity in our HAT assay, we assessed H4 acetylation in a time course. H4K16ac was already detectable after 2 min and the signal increased over the course of 90 min, when saturation was reached (Figure 1B and Supplementary Figure S1E). The H4K12ac signal was detected after 30 min and also reached saturation at 90 min. We thus defined the linear range between 5 and 60 min incubation time.

We concluded that our 4-MSL complexes are active HATs that efficiently acetylate histone H4 in nucleosome arrays. Contrary to our expectation, we also detected H4K12 acetylation, which is at odds with the prevalent view of MOF activity in the context of *Drosophila* dosage compensation. Early staining of polytene chromosomes with specific antibodies had shown that the H4K16ac mark, but not H4K12ac, is enriched on the X chromosome in male larvae (64). The *in vitro* result could be due to a hitherto unappreciated lack of MOF specificity in the 4-MSL complex, or due to cross-reactivity of the H4K12ac antibody. To clarify this point, we applied a mass spectrometry approach, in which each histone acetylation can be individually detected and quantified in combinatorial patterns (27).

### The 4-MSL complex acetylates lysines along the H4 tail upon extended incubation

Mass spectrometry (MS) enables very sensitive detection of peptides bearing multiple, combinatorial modifications. Because acetylated peptides are detected along with the corresponding unmodified peptide, the relative occurrence of each modification can be determined. Mindful of variability and batch effects, each experiment involved parallel reactions with three independent enzyme preparations, and was repeated at least once.

**Figure 2.**
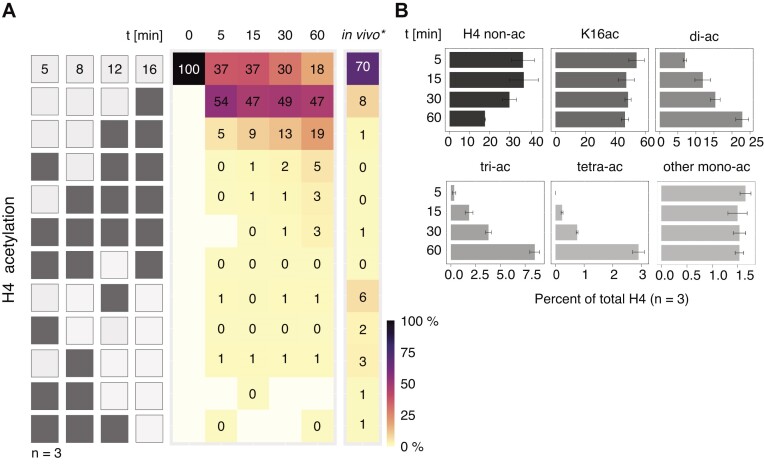
The 4-MSL complex progressively acetylates H4 tail lysines *in vitro*. (**A**) Heatmap displaying H4 tail lysine acetylation on a nucleosome array substrate by 50 nM 4-MSL complex as quantified by mass spectrometry. The left panel displays combinatorial acetylation motifs on H4 lysines 5, 8, 12 and 16. Light shading displays non-acetylated residues, while dark shading means acetylation at corresponding position. The map shows all combinations that can be measured by MS2 and thus lacks some diacetylated forms. The mean abundance of acetylated motifs at indicated time points is presented. First row shows the mean levels of non-acetylated H4 at different conditions. *N* = 3 independent 4-MSL preparations. Motifs falling below the detection limit of 0.003% are indicated in white. ‘0%’ refers to values < 0.5%, but above detection limit. For comparison, the levels of H4 tail lysine acetylation in male S2 cells are displayed (‘*in vivo’*) (27). (**B**) Bar plot summarizing the abundance of mono- and oligo-acetylated H4 tail motifs detected in (A). Grey shading indicates different scales. ‘di-/tri-ac’ represents the sum of all possible di- or tri-acetylated H4 tail motifs. ‘tetra-ac’ refers to the fully acetylated H4 tail. ‘other mono-ac’ cumulates levels of K5ac, K8ac and K12ac. Standard error of the mean of 3 independent 4-MSL preparations is given.

The acetylation reactions were set up according to the conditions determined in the pilot experiments and subjected to MS analysis, as before (27). The unmodified H4 peptide G4-R17 and the control peptides for H3 (K18-R26 and Y41-R49) were reliably detected. Our targeted approach quantifies H4 peptides that are monoacetylated at any of the four lysines (K5, K8, K12, K16), any combination of di- or tri-acetylation as well as the tetra-acetylated form (Figure 2A). The amount of each peptide species was determined by the corresponding MS1 peak area, considering the number of light- and heavy-isotope acetyl groups (Supplementary Figure S2A). Individual motif intensities were quantified by targeted MS scan. We restricted ourselves to mapping those diacetylated motifs that can be readily identified by MS1/MS2 (Figure 2A). Each enzymatically acetylated peptide species is expressed as fraction of the sum of all H4 peptides (unmodified and modified), determined by the summed up MS1 areas under all relevant peaks (27).

In absence of the 4-MSL complex (0 min) no acetylation was detected on the recombinant histones (detection limit 0.003%) (Figure 2A). Impressively, the level of monoacetylated H4K16 reached on average 54% already during 5 min, whereas monoacetylation of K5, K8 or K12 remained at or below 1% and did not change during the incubation time (Figure 2A, B). This result documents the selectivity of the 4-MSL complex to modify K16. However, as the reaction continued, the K16 mono-acetylation did not increase further, but rather decreased in favor of di-acetylation, which contained both K12ac and K16ac. Curiously, tri- and tetra-acetylated peptides increased at later time points (Figure 2A, B). Tri-acetylated peptides always contained K16ac and K12ac. Overall, combinations lacking K16ac were scarce (1%) and most of them were below the detection limit <0.003% (Figure 2A).

These results are consistent with the earlier finding that H4K12ac was detected by Western blot after 30 min (Figure 1B). The MS analysis shows that K12 is indeed acetylated, but in the form of K12K16 di-acetylation, which averages 13% after 30 min. Accordingly, the continued increase of the K16ac signal in the Western blot upon longer incubation times is explained by the accumulation of combinatorial acetylation patterns including K16ac, which are distinguished by mass spectrometry.

### MOF is a processive enzyme

The finding that all measured combinations of H4 tail acetylation contain K16ac suggests that the K16 site was primarily recognized by the enzyme. This finding resonates with earlier observations of H4 tail acetylation patterns in butyrate-treated HeLa cells, which had led to the suggestion of a ‘zip’ model of directional, progressive H4 tail acetylation starting at K16 and moving ‘outwards towards the N-terminus (31).

**Figure 3.**
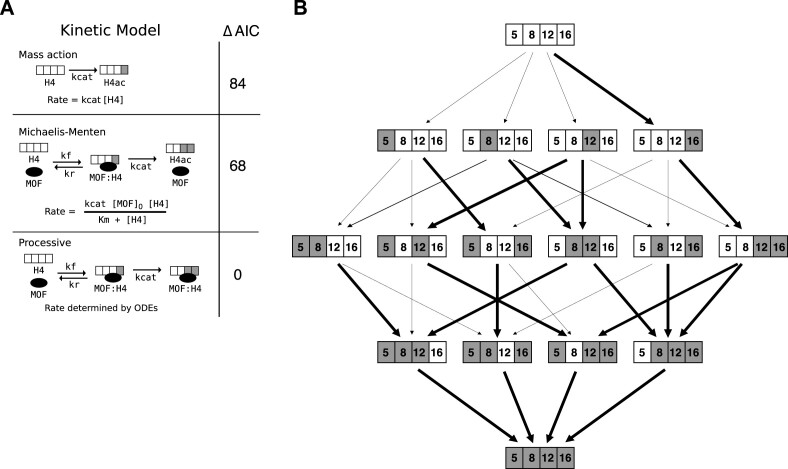
H4 tail acetylation kinetics by MOF is described by a processive model. (**A**) Tested hypothetical scenarios for MOF kinetics: The ‘mass action’ model assumes that the reaction is not processive and that the rate of acetylation depends on the current concentration of the substrate (i.e. enzyme is in excess). The ‘Michaelis–Menten’ model assumes that the reaction is not processive and that the enzyme-substrate complex concentration is at equilibrium throughout the experiment. The ‘processive’ model explicitly models the enzyme, substrate, and product interactions in a system of ordinary differential equations (ODEs), and does not have the enzyme-substrate complex equilibrium assumption of the Michaelis-Menten model. The processive model does not assume that the enzyme is in excess. The rate in the processive model is implicitly determined by a system of ODEs (Supplementary Note S2). Models were calibrated to the time-dependent H4 tail acetylation data (Figure 2). The difference in the Akaike information criterion (ΔAIC) relative to the AIC of the best model, is shown for each model. A lower AIC suggests that a model is better supported by the data. (**B**) Relative probabilities for site-specific acetylation of each motif on wild-type nucleosome arrays. The ensemble used in Supplementary Figure S3A and S3B contains samples of the acetylation catalysis constants ‘kcat’ (see (A), ‘Processive’). Black arrows represent the relative probability for a particular motif to be acetylated at a particular site. The thickness of each black arrow is the ensemble's median value for the corresponding reaction rate constant. The thick black arrows indicate the highest probability reaction for that motif, to which the thickness of other arrows coming from the same motif is normalized.

Such a ‘zip’-like mode of H4 tail acetylation would possibly require a processive MOF enzyme. To investigate the kinetics of MOF activity we applied mathematical modeling, testing the following hypotheses (Figure 3A): (i) acetylation of H4 is not processive and the enzyme is available in excess (‘mass action’) (55). (ii) Acetylation of H4 is not processive and the enzyme is not available in excess (‘Michaelis–Menten’). (iii) Acetylation of H4 occurs processively, assuming the enzyme stays bound to the substrate to acetylate another lysine residue (‘processive’).

The models were calibrated to the time-dependent H4 tail acetylation data (Figure 2) and compared using the Akaike Information Criterion (ΔAIC) (Figure 3A). Comparing the hypotheses, the processive model was most supported by the data, followed by the Michaelis-Menten model. Further, a processive model assuming that the transition of the enzyme to another nucleosome array takes longer than a transition within an array was most supported by the data, suggesting that there is a spatial effect in the enzyme behavior.

To assess motif-specificity of the enzyme, we performed a Bayesian uncertainty quantification (Figure 3B, Supplementary Figure S3A). The assessment of the relative acetylation rates for different motifs reveals that the acetylation sequence of H4 → H4K16 → H4K12K16 accounts for much of the flux. However, in the uncommon instances where the first acetylated lysine is one of K5, K8 or K12, then the second acetylated lysine is often not K16 either (Figure 3B, e.g. H4K12 → H4K5K12 and H4K12 → H4K8K12). Overall, the model supports the experimental data of a selective K16 acetylation by the 4-MSL complex over other acetylation sites.

### Exploring the requirement of lysine 16 for H4 tail acetylation by 4-MSL

**Figure 4.**
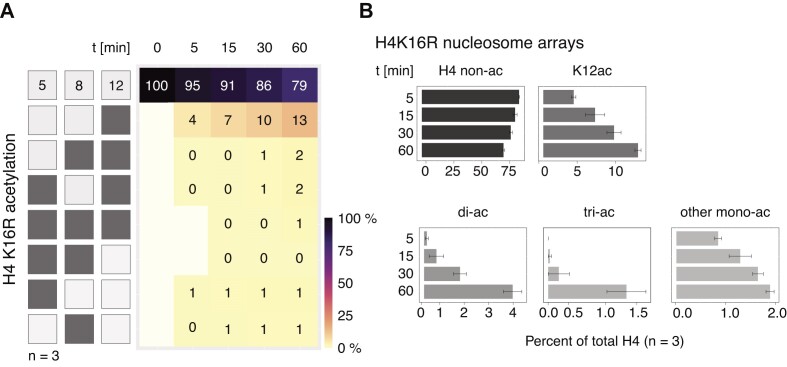
The 4-MSL complex can acetylate H4K12 in H4K16R mutant nucleosome arrays. (**A**) Heatmap showing H4 tail lysine acetylation levels as in Figure 2A using 50 nM 4-MSL complex and nucleosome arrays bearing the H4K16R mutant as substrate. The left panel displays combinatorial acetylation motifs on H4 lysines 5, 8 and 12. Light shading displays non-acetylated residues, while dark shading means acetylation at corresponding position. The mean abundance of acetylated motifs at indicated time points is presented. First row shows the mean levels of non-acetylated H4 at different conditions. *N* = 3 independent protein preparations. Motifs falling below the detection limit of 0.003% are indicated in white. ‘0%’ refers to values <0.5%, but above detection limit. (**B**) Bar plot summarizing the abundance of mono- and oligo-acetylated H4 tail motifs detected in (A). Grey shading indicates different scales. ‘di-ac’ represents the sum of all possible di-acetylated H4 tail motifs. ‘tri-ac’ refers to the fully acetylated H4 tail in absence of K16. ‘other mono-ac’ cumulates levels of K5ac and K8ac. Standard error of the mean of three independent 4-MSL preparations is given.

The main implication of this model is that acetylation of the H4 tail at K16 is the prerequisite for any further acetylation at more N-terminal lysines. To address this hypothesis, we assembled nucleosome arrays with a mutant H4, in which K16 was replaced by arginine (H4K16R). Partial MNase digestion demonstrated that the quality of the mutant arrays was similar to wild-type arrays (Supplementary Figure S1B). Parallel acetylation reactions of mutant and wild-type arrays were analyzed by Western blotting (Supplementary Figure S2C). No signal was detected with the H4K16ac-specific antibody, confirming its substrate selectivity. The K12ac-antibody detected a weak signal at longer incubation times. Consistent with this, MS quantification showed that mono-acetylation of K12ac was most abundant, reaching on average 13% after 60 min (Figure 4A, B and Supplementary Figure S2B). Di- and tri-acetylations were rare (maximally 2%) but showed a time-dependent increase.

The ensemble of samples was used to predict the acetylation dynamics assuming that all reactions involving K16 are disabled (Supplementary Figure S3B). The simulation suggests that MOF acetylates K12 at a higher rate in the absence of K16; a prediction that is validated by the experimental data obtained with H4K16R mutant nucleosome arrays (Figure 4). However, the simulation also predicted higher rates of K8ac and K5ac alone or in combination with K12ac, which was not observed in the experiments.

Considering the significant acetylation of K12 upon extended incubation, the requirement of K16 as a ‘point of entry’ is not absolute: the 4-MSL complex can recognize the K16R histone tail as substrate, but the reaction is much less efficient. Interestingly, the 4-MSL complex still preferentially acetylates the most ‘internal’ lysine, in accord with the ‘zip’ model.

### RNA improves the specificity of 4-MSL complex towards H4K16 mono-acetylation

The observation of abundant oligo-acetylation *in vitro* is in stark contrast with the *in vivo* situation, where these patterns are rare. In *Drosophila* S2 cells, 70% of the H4 tails is found non-acetylated and the most abundant acetylation is the K16 mono-acetylation (8%, Figure 2A, right panel). In cells, low-level monoacetylation of K12, K8 and K5 are detected, which are placed by other acetyltransferases such as KAT1, KAT5 (Tip60) and KAT7 (chameau) (27). However, di-, tri- and tetra-acetylations are rare (<1%, Figure 2A, right panel) (27).

It is possible that ubiquitous HDACs restrict the occurrence of oligo-acetylation of the H4 tail in cells (31). Alternatively, a regulatory factor may be missing in our *in vitro* assay, which improves the selectivity of MOF in the context of the DCC in male cells. A candidate for such a factor is the male-specific long, non-coding RNA *roX*, which is essential for the functioning of the DCC (16,37–39). Conceivably, the RNA might boost the acetylation reaction. To explore a role for *roX2* in the regulation of H4 acetylation, the *in vitro-*transcribed RNA was included in the HAT assay at different molar ratios relative to the 4-MSL complex. The assay was performed in presence of ATP and an equimolar ratio (with respect to 4-MSL) of the RNA helicase MLE, which is required for *roX2* incorporation into the DCC (41). The MS quantification revealed that the RNA did not stimulate the HAT reaction, since the levels of H4K16ac remained constant (Supplementary Figure S4A, B). Instead, RNA led to the reduction of di-, tri- and tetra-acetylation and the relative prevalence of the specific H4K16ac motif increased (Supplementary Figure S4A, B). The suppression of the oligo-acetylation patterns was independent of MLE, as addition of MLE did not impact the acetylation distribution or the RNA effect (Supplementary Figure S4A, B).

**Figure 5.**
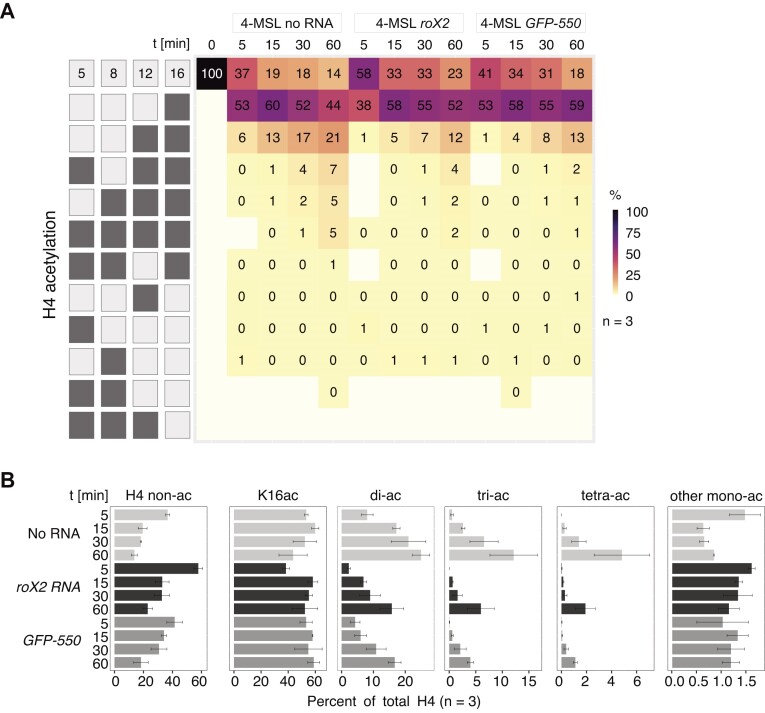
RNA suppresses oligo-acetylation of the H4 tail by the 4-MSL complex *in vitro*. (**A**) Heatmap displaying H4 tail lysine acetylation by 50 nM 4-MSL complex in absence or presence of 50 nM MLE and a 2-fold molar excess of *roX2* and *GFP-550* RNA, respectively, at indicated time points. All reactions contained 1 mM ATP. The left panel displays combinatorial acetylation motifs on H4 lysines 5, 8, 12 and 16. Light shading displays non-acetylated residues, while dark shading means acetylation at corresponding position. The map shows all combinations that can be measured by MS2 and thus lacks some diacetylated forms. First row shows the mean levels of non-acetylated H4. *N* = 3 independent protein preparations. Motifs falling below the detection limit of 0.003% are indicated in white. ‘0%’ refers to values < 0.5%, but above detection limit. (**B**) Bar plot summarizing the abundance of mono- and oligo-acetylated H4 tail motifs detected in (A). ‘di-/tri-ac’ represents the sum of all possible di- or tri-acetylated H4 tail motifs. ‘tetra-ac’ refers to the fully acetylated H4 tail. ‘other mono-ac’ cumulates levels of K5ac, K8ac and K12ac. Standard error of the mean of three independent 4-MSL preparations is given.

To test whether this effect was specific, *roX2* RNA was compared to an *in vitro*-transcribed *GFP* RNA of matching size (*GFP* 550 nt, *roX2* 552 nt (41)), which shares no sequence similarity with *roX2*. In this experiment we kept the total mass of RNA constant, but assayed the development of the acetylation pattern in a time course (Figure 5A, B). We observed that both RNAs suppressed oligo-acetylation equally well, while the mean level of H4K16ac remained relatively constant. Therefore, we conclude that the effect of RNA on H4 tail acetylation by MOF *in vitro* is largely non-specific. Interestingly, equal masses of single-stranded DNA (*poly-dA*, 250–500 nt) and tRNA were inactive (Supplementary Figure S4A, B), hinting at a requirement for RNA of a certain length rather than non-specific polyanions to suppress oligo-acetylation of the H4 N-terminus.

**Figure 6.**
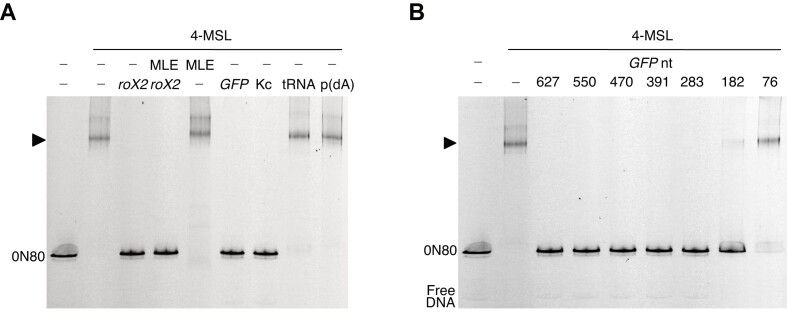
Interaction of the 4-MSL complex and mononucleosomes is destabilized by long RNA. (**A**) RNA non-specifically destabilizes the 4-MSL-mononucleosome interaction as demonstrated by electrophoretic mobility shift assay (EMSA). EMSA was performed with 50 nM 4-MSL and 10 nM 0N80 mononucleosomes in absence or presence of MLE (50 nM), *roX2* RNA (50 nM), *GFP-550* RNA (50 nM), total RNA from *Drosophila* Kc cells (200 ng), yeast tRNA (200 ng) and poly(dA) polymer (200 ng), respectively. (**B**) Disruption of 4-MSL-mononucleosome complexes is RNA length-dependent. EMSA was performed with 50 nM 4-MSL and 10 nM 0N80 mononucleosomes in absence or presence of *GFP* RNA variants of indicated length (in bases) at a concentration of 50 nM each.

To explore the underlying mechanism of RNA-mediated suppression of oligo-acetylation, we tested whether RNA affects nucleosome binding of the 4-MSL complex. Towards this end, we performed electrophoretic mobility shift assays (EMSAs) with nucleosomes assembled on a DNA containing a ‘Widom 601′ nucleosome positioning sequence. The nucleosomes were positioned asymmetrically on this DNA with an 80 bp linker DNA on one side (0N80). In absence of RNA the MSL complex readily and stably bound these nucleosomes, indicated by a clearly retarded band shift in the EMSA. Surprisingly, the 4-MSL–nucleosome complex was dissociated upon addition of 25–100 nM of either *roX2* RNA (552 nt) or *GFP* RNA (550 nt) (Supplementary Figure S4C). Further experiments demonstrated that this disruption was independent of the presence of MLE/ATP and was also observed with total RNA extracted from *Drosophila* Kc cells (heterogenous RNA of various sizes, but lacking *roX*) (Figure 6A). Strikingly, neither yeast tRNA (70–90 nt long) nor single-stranded DNA (poly-dA) were effective. Exploring the length-dependence of complex disruption, we found that *GFP* RNAs above 180 nt were effective and 76 nt RNA did not disrupt the enzyme-substrate complex. A partial effect around 180 nt length suggested that RNA should be longer than 180 nucleotides to be effective (Figure 6B).

The similar effects of diverse RNA types on the ability of the 4-MSL complex to stably bind nucleosome substrates in an EMSA and to suppress oligo-acetylation of the H4 tail leads us to hypothesize that RNA may reduce the dwelling time of the enzyme on the nucleosome thereby limiting the time for processive acetylation of multiple lysines.

### RNA specifically affects the HAT activity of MOF, but not dTip60

The non-specific effect of RNA on the HAT activity of the 4-MSL complex could either be due to charge interactions between the polyanion and positively charged amino acids on the nucleosome (notably the lysines of the H4 tail). Alternatively, the RNA could allosterically affect the 4-MSL complex to increase the specificity of MOF towards lysine 16. If the nucleosome substrate was occluded, different HAT enzymes may be similarly affected. To address this issue, we assessed the effect of RNA on another MYST HAT of *Drosophila melanogaster*, dTip60 (KAT5), which has a known selectivity for H4K12 (11,27). Like MOF, Tip60 alone is unable to acetylate nucleosomal H4 (15,34).

Inspired by the example of yeast, where the evolutionary conserved HAT, Esa1, is functional in a trimeric complex termed ‘piccolo NuA4’ (15,34), we expressed *Drosophila* dTip60 along with the corresponding fly subunits E(Pc) and ING3 (11). We term the resulting trimeric HAT complex ‘dTIP60^piccolo^’ since the trimeric TIP60 assembly *in vivo* resides in the much larger DOMINO-A complex (65). The dTIP60^piccolo^ complex was expressed from a multi-bac vector in Sf21 cells and purified (Supplementary Figure S5A). The presence of the three subunits was verified by mass spectrometry.

**Figure 7.**
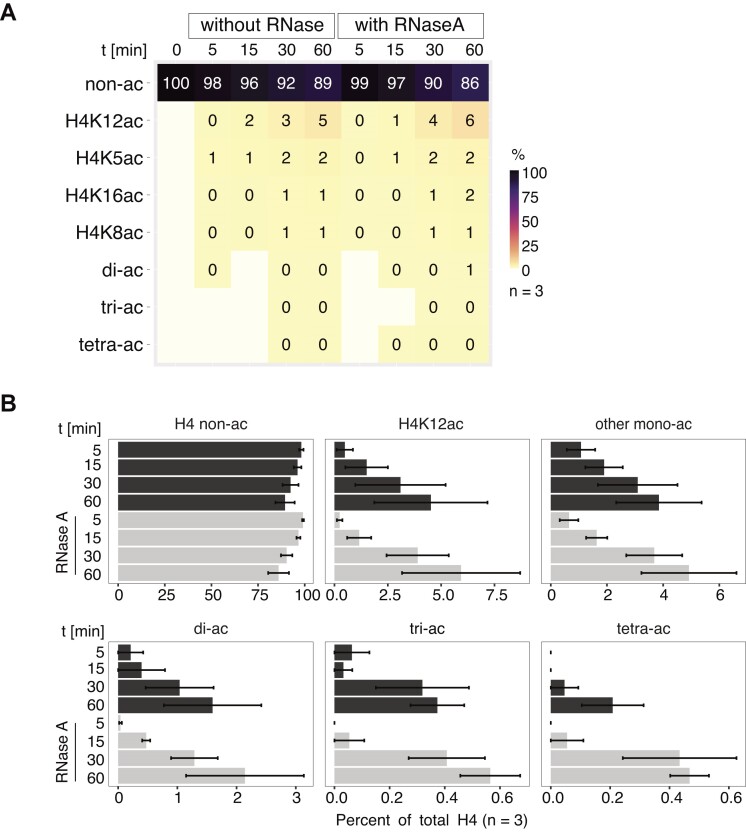
The histone acetyltransferase activity of the dTIP60^piccolo^ complex is not influenced by RNA. (**A**) Heatmap showing the abundance of H4 tail acetylation by 50 nM dTIP60^piccolo^ on nucleosome arrays at indicated time points as detected by mass spectrometry. Reactions were performed in absence or presence of RNase A. ‘di-/tri-ac’ represents the sum of all possible di- or tri-acetylated H4 tail motifs. ‘tetra-ac’ refers to the fully acetylated H4 tail. *N* = 3 independent protein preparations. Motifs falling below the detection limit of 0.003% are indicated in white. ‘0%’ refers to values < 0.5%, but above detection limit. (**B**) Bar plot summarizing the abundance of mono- and oligo-acetylated H4 tail motifs detected in (A). ‘di-/tri-ac’ represents the sum of all possible di- or tri-acetylated H4 tail motifs. ‘tetra-ac’ refers to the fully acetylated H4 tail. ‘other mono-ac’ cumulates levels of K5ac, K8ac and K16ac. Standard error of the mean of 3 independent protein preparations is given.

HAT-MS assays with dTIP60^piccolo^ revealed a pronounced selectivity of H4K12 over other mono-acetylations, including H4K16 (Figure 7A, B). Although the overall HAT activity was much weaker than that of the 4-MSL complex, the sensitivity of the MS approach allowed to follow the increase in acetylation to reach 5% K12ac during the 60-min time course (Figure 7A, B). In remarkable contrast to the 4-MSL reaction, H4K5 was mono-acetylated to an average of 2% in addition to H4K12ac, suggesting the absence of a ‘zip’-type mechanism. The levels of all di- or oligo-acetylated peptides together was below 2%. This finding shows that the two MYST family HATs approach the nucleosome substrate in very different ways.

We were concerned that the low HAT activity of several independent dTIP60^piccolo^ preparations was due to contaminating RNA and indeed found that the preps contained abundant RNAs despite RNase A and benzonase treatments during the purification (Supplementary Figure S5B). In contrast, 4-MSL preparations are free of RNA (not shown). Inclusion of RNase A during the HAT assay led to complete degradation of the RNA contaminants (Supplementary Figure S5B), yet did not change the acetylation efficiency or specificity (Figure 7A, B). To ensure that we would score any repressive effect of RNA that we found in 4-MSL reactions, we further added an excess of *GFP* RNA to the HAT reactions (Supplementary Figure S5B). Neither the addition of RNA, nor the complete removal of RNA from the preparations changed the activity or specificity of the dTIP60^piccolo^ (Supplementary Figure S5C).

We conclude that the influence of RNA on the acetylation of the H4 N-terminus is a particular feature of the 4-MSL complex and not a property shared between the two MYST HAT family members, MOF and dTip60 (66).

## Discussion

The acetylation of lysine 16 of histone H4 (H4K16ac) disrupts the interaction between the ‘basic patch’ in the N-terminus of histone H4 and the acidic patch formed by H2A-H2B on near-by histone octamers, thus unfolding of the nucleosome fiber. This direct effect on chromatin opening and transcription activation contrasts the indirect effect of other histone tail acetylations, which typically function through recruitment of bromodomain-containing regulators.

In *Drosophila*, H4K16 acetylation is catalyzed by MOF (KAT8) in the context of the male-specific lethal dosage compensation complex (DCC) and the ubiquitous NSL complex (1,67). In the DCC, MOF is thought to acetylate H4K16 with high specificity, a feature that appears to be conserved in human MSL complexes (19,27). However, as part of the NSL complex, MOF has been reported to acetylate H4K5, K8 and K12 (19,22). This notion is supported by a systematic analysis of KAT activities in female *Drosophila* cells, which showed that depletion of the key NSL subunit NSL1 leads to reduced H4K5ac (and statistically not robust K8ac) in addition to lower H4K16ac (27). These findings reveal that the selectivity of MOF to acetylate H4 tail lysines is modulated by molecular context.

To explore the molecular basis of the exquisite H4K16ac specificity in dosage compensation, we generated core MSL complexes from recombinant subunits (4-MSL). Using specific antibodies, we observed that the 4-MSL complex dominantly acetylated H4K16, but also detected H4K12 acetylation. This unexpected finding and concerns about cross-reactivity of Kac-antibodies triggered a systematic analysis of the acetylation reaction using a targeted MS workflow (27).

### Progressive and processive H4 oligo-acetylation by MOF in the 4-MSL complex

Mass spectrometry enables the sensitive and quantitative description of site-specific acetylation, including combinatorial patterns of two or more modifications. Monitoring the acetylation of the H4 tail, we found that the 4-MSL complex quickly acetylated K16 with exquisite specificity. However, as time proceeded, patterns of di-, tri- and even tetra-acetylation emerged. Remarkably, all combinatorial acetylation patterns contained H4K16ac, suggesting that this lysine is first acetylated followed by additional modification of the K16-acetylated tail. In fact, mathematical modeling testing different hypothesis suggests that MOF is a processive enzyme, explaining the H4 acetylation patterns observed *in vitro*.

Our findings are reminiscent of the ‘zip’ model of Burlingame and colleagues who concluded from their analysis of H4 tail acetylation in butyrate-treated HeLa cells that H4 tail acetylation proceeds from the most ‘internal’ lysine (closest to the nucleosome body) outwards to the N-terminus (31). In their case, the development of oligo-acetylated forms resulted from KDAC inhibition and thus involved an ‘outside-in’ gradient of deacetylation. We now show that processive ‘zip’-type acetylation is an intrinsic property of the 4-MSL complex. Upon mutation of K16R, the overall acetylation is diminished, but the residual motifs now dominantly contain K12ac, which was also predicted by mathematical modeling.

The data suggest that the initial substrate recognition of the nucleosome involves interactions of the 4-MSL complex with nucleosome features in addition to the H4 tail, such as histone surfaces and/or DNA. These aspects of substrate recognition should be contributed by MSL subunits other than MOF, since MOF alone cannot acetylate nucleosomal H4 (63).

### Histone acetylation selectivity depends on molecular context

Histone acetylation patters in cells arise from the complex interplay of numerous KAT and KDAC activities (27). Abundant oligo-acetylations of the H4 tail accumulate upon general inhibition of histone deacetylases, revealing the involvement of these ubiquitous enzymes in keeping overall acetylation at optimal level for gene regulation and genome stability (27,31). Our defined *in vitro* reactions inform about the intrinsic ability of the 4-MSL complex to acetylate the histone H4 tail. Such information is important to begin disentangling the complexity of acetylation dynamics *in vivo*.

Clearly, MOF in the context of 4-MSL is able to acetylate H4 tail lysines in addition to K16. It is possible that the development of oligo-acetylation is a consequence of the low-complexity reaction conditions, including non-physiological, high enzyme concentrations and the absence of competing interactors with the nucleosome substrate. Although the local concentrations of the DCC and its cofactor acetyl-coenzyme A at target chromatin are difficult to know, we note that in cells about 70% of the H4 tail peptide is unmodified at steady state, showing that acetylation reactions are limited (27).

Considering the substrate selectivity of MOF in cells, one must distinguish between female and male cells. Female cells only contain the NSL complex (67). We found earlier that female Kc cells contain combinatorial H4 acetylation pattern with the following abundances (27): 16ac (3.3%), 16ac12ac (0.8%), 16ac8ac (0.4%), 16ac12ac8ac (0.3%), 16ac8ac5ac (0.13%), tetra-ac (0.3%). Upon depletion of MOF several of the combinatorial marks decreased. It is, therefore, possible that in the context of the NSL complex an intrinsic, relaxed substrate selectivity of MOF is observable. Alternatively, MOF only acetylates K16 and the additional acetylation marks are contributed by other KATs. The recent finding that the NSL complex predominantly acetylates K5, K8 and/or K12 (and not K16) in human cells suggests more complex scenarios (19). The complexity might be further increased by the interplay between KATs and KDACs, which *in vivo* are abundant and might erase surplus acetylation patterns.

In male *Drosophila* cells, MOF resides in the MSL-DCC in addition to the NSL complex, leading to higher levels of K16 monoacetylation (27). The DCC mainly acetylates nucleosomes in transcribed chromatin. Transcribed chromatin is broadly marked by methylation of H3K36, which is recognized by a reader subunit in the DCC, MSL3. Our *in vitro* reaction with recombinant, unmodified nucleosomes shows that the methyl-mark is not required for efficient acetylation of H4. The formal possibility that the H3K36 methylation improves the specificity of acetylation of K16 monoacetylation needs to be tested in future experiments.

Because the 4-MSL complex dominantly acetylates only K16 at short incubation times, we speculate that *in vivo* the dwelling time of the enzyme on any individual nucleosome is never long enough to proceed beyond K16 monoacetylation. Because the DCC *in vivo* prominently differs from the recombinant 4-MSL complex we assayed *in vitro* by the presence of non-coding *roX* RNA, we explored the possibility that RNA may modulate the outcome of the acetylation reaction.

### Inclusion of RNA reduces acetylation processivity and improves the specificity for K16ac

We found that including RNA of the size of *roX2* (550 nt) in the HAT assay dampened the reaction, leading to reduced levels of di- and oligo-acetylation. As a result, more H4 tails remained unacetylated and the relative fraction of H4K16 mono-acetylation increased, a situation that approaches the physiological state. We also observed that such RNA destabilized the 4-MSL-nucleosome complex during electrophoresis. We speculate that the presence of RNA may increase the off-rate of MOF-nucleosome binding and that the reduced dwelling time may assure predominant H4K16 mono-acetylation.

Of course, it is possible that the RNA effect is an artefact of the *in vitro* reaction. The polyanion may bind to the positively charged substrate lysines, leading to steric hindrance of the enzyme. However, the fact that deoxyadenosine polymers as well as short RNAs did not affect the HAT reaction of the 4-MSL complex argues against a purely electrostatic effect. Furthermore, we ruled out this scenario by assaying a second MYST HAT complex, the dTIP60^piccolo^ complex. The acetylation showed a preference for K12, in line with the known *in vivo* substrate selectivity of dTip60 (68), but it was clearly not affected by RNA. In fact, dTip60 was shown to bind pre-mRNAs in the *Drosophila* brain (66), a characteristic reflected by the vast amount of RNA that co-purified with the recombinant dTIP60^piccolo^ complex. Despite its RNA-binding potential and in contrast to the MSL-DCC, the enzymatic activity and/or the nucleosome binding mode of dTIP60^piccolo^ are supposedly not regulated by association with RNA. dTip60 functions in the larger DOMINO complex *in vivo*, which contains many more subunits (11). One of these subunits is Eaf6 (Esa1-associated factor 6), which is a stabilizing factor for the yeast Piccolo complex (69–71). The relatively low enzymatic activity scored by the dTIP60^piccolo^ complex, might indicate an important role of *Drosophila* Eaf6 for efficient acetylation.

An influence of RNA on properties of the 4-MSL complex is not unexpected, given that all subunits can bind RNA (33,72–74). On the other hand, the effect of RNA was clearly non-specific: an irrelevant sequence encoding GFP worked as well as *roX2* RNA. Could this have a physiological relevance?

We envision several scenarios. First, the association of the DCC with transcribed chromatin may be disrupted non-specifically by nascent RNA. The RNA associated with the transcription machinery might feed forward to assure the unhindered progress of the elongating RNA polymerase by displacing chromatin-bound DCC. At the same time, this would promote the turnover of the complex with chromatin sites, such that different sites will be sampled and the acetylation will spread more widely. There is ample precedent for such a scenario. MSL2 has been shown to bind not only to *roX* RNA, but to 120 nascent transcripts from the X chromosome in close proximity of MSL2 binding sites (75). Moreover, nascent RNA has been shown to disrupt the interactions of many epigenetic regulators with chromatin (76,77), including polycomb complexes (78–83). Recently, it was shown that G-quadruplex RNA induces a PRC2 dimer, in which the nucleosome binding domain is occluded (84). Another example is dissociation of NELF from paused polymerase II elongation complexes by enhancer-derived RNAs (eRNAs) (85). Because the DCC selectively modifies active chromatin, encounters with nascent RNA are likely.

More generally, RNA emerges as an abundant component of interphase chromatin independent of its transcription status. Diverse chromatin-associated RNAs have been shown to prevent chromatin condensation and thus to ensure its functionality (86–88). Although the consequences of crude RNase-treatment of nuclei should be interpreted with caution (89–91), many chromatin regulators can bind RNA and their chromatin interactions may well be affected non-specifically by ubiquitous and abundant RNAs.

Our finding of non-specific effects of RNA on the processivity of histone acetylation by MOF does not exclude a specific role of *roX*. The association of *roX2* RNA with MSL proteins is catalyzed by the helicase MLE (33,41,92). ‘Incorporation’ of the RNA relies on evolutionary conserved features in the 3′ end of *roX* and requires direct association with MSL1 and MSL2 (33,41,92). In fact, Ilik et al. (92) suggested that the binding of *roX* to MSL2 competes for its chromatin association. Accordingly, MLE-dependent remodeling of *roX* constitutes a switch that assures turnover of the DCC in chromatin to prevent non-productive trapping and to promote spreading of H4K16 acetylation. However, *roX* RNAs are long and most of their sequences are not conserved. We envision that some aspects of its function may not require a defined RNA sequence or structure, but rather rely on high local concentration of RNA close to MSL proteins, a parameter that can be tuned by the helicase MLE. In support of such a scenario, RNA concentrations that led to complete dissociation of the 4-MSL complex with nucleosomes did not inhibit the HAT reaction, but rather limited the non-physiological progression to H4 oligo-acetylation.

Our *in vitro* study informs about the intrinsic properties of defined components in a low-complexity system. Further efforts must be devoted to understanding the role of specific and non-specific effects of RNA on the functions of the DCC in a physiological setting.

## Supplementary Material

gkae123_Supplemental_File

## Data Availability

The mass spectrometry proteomics data have been deposited to the ProteomeXchange Consortium via the PRIDE (93) partner repository with the dataset identifier PXD046636. R script for calculation of PTM levels determined by mass spectrometry is provided in Supplementary Note S3. Scripts and data for mathematical modeling are provided in Supplementary Note S2 and have been deposited at Zenodo (DOI: 10.5281/zenodo.10221453).
