# Supplementary material for: Processivity and specificity of histone acetylation by the male-specific lethal complex
Source: Nucleic Acids Res. 2024 Feb 26;52(9):4889–905. doi: 10.1093/nar/gkae123 (PMC11109948; doi:10.1093/nar/gkae123)
Supplement: gkae123_Supplemental_File [file gkae123_supplemental_file.pdf]

## Supplementary Figure 1

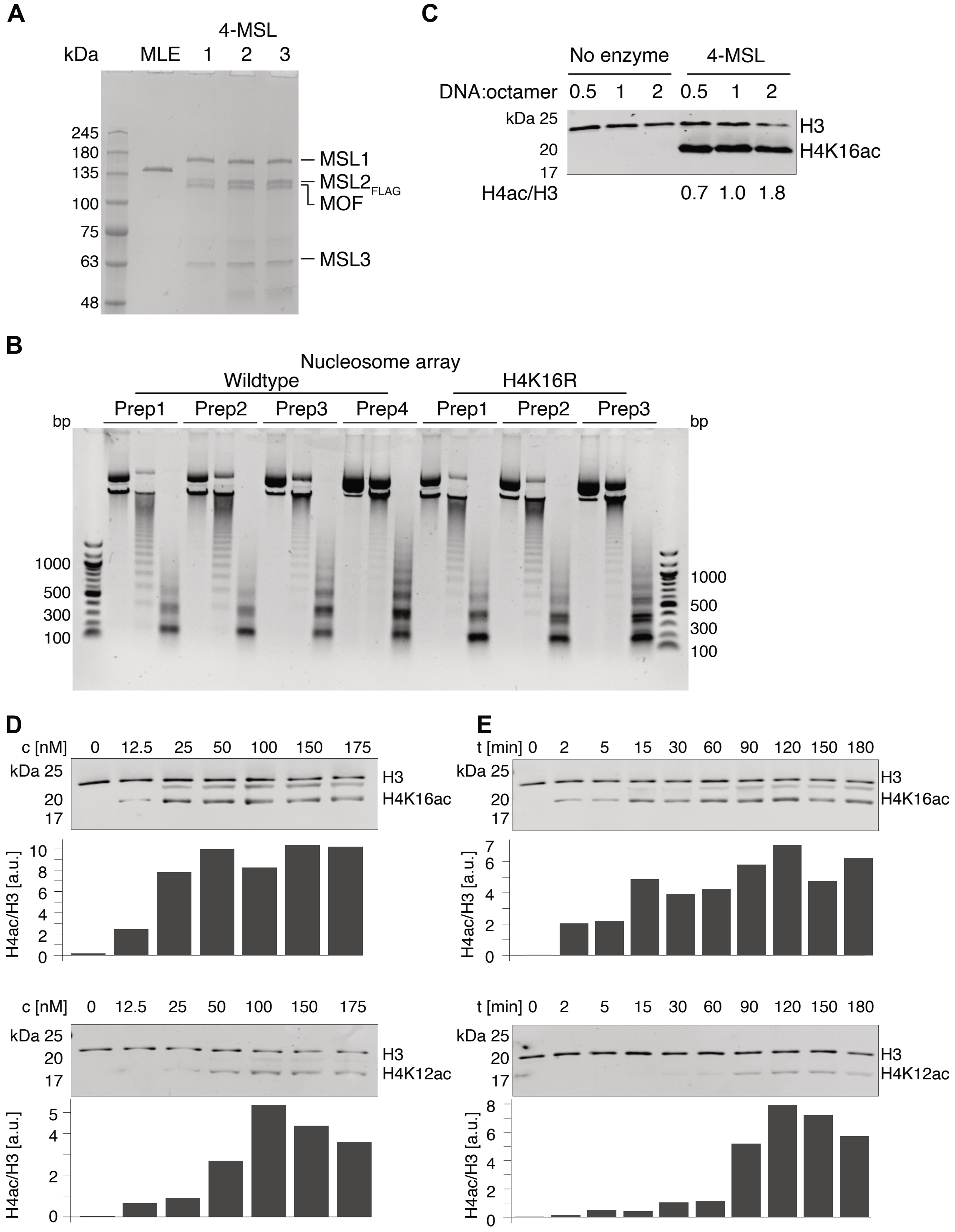


Supplementary Figure S1: The 4-MSL complex acetylates H4K16 and H4K12 on a nucleosome array substrate *in vitro*.

(A) Coomassie-stained SDS-PAGE showing FLAG-affinity-purified MLE (helicase 'maleless') and three independent preparations of the 4-MSL complex (MSL1:MSL2-Flag:MOF:MSL3).

(B) Quality control of 4 wild type (WT) and 3 histone H4K16R mutant nucleosome array substrates, respectively, assembled by salt gradient dialysis. The arrays were treated with micrococcal nuclease for 30 s, 60 s or 5 min and purified DNA fragments representing the 'nucleosome

ladder' were visualized on a 1.5% agarose gel stained with Midori green. Note that double bands arise due to different repeat lengths on the vector backbone (147 bp) and on the 25 x Widom 601 repeat insert (197 bp).

(C) Histone acetylation assay with the 4-MSL complex and nucleosome arrays of different assembly degree. Plasmid DNA and histone octamers were assembled at a molar ratio of 0.5, 1.0 and 2.0, respectively, and incubated with 50 nM 4-MSL complex for 60 min. Acetylation of histone H4 was detected by Western blot using an antibody specific for H4K16a. H4 acetylation levels relative to histone H3 are indicated, the 1:1 ratio arbitrary value was set to 1.0 and used as a normalization for the other two assembly degrees.

(D) Independent replicate of the histone acetyltransferase assay presented in Figure 1A, using purified 4-MSL complex at indicated concentrations (c) and a nucleosome array substrate. Incubation time was 60 min. Acetylation of histone H4 was detected by Western blot using antibodies specific for H4K16ac and H4K12ac, respectively. Histone H3 served as loading control and was used to quantify relative H4 acetylation as represented in the bar plot as arbitrary units (a.u.).

(E) Independent replicate of the histone acetylation assay presented in Figure 1B, using 50 nM 4-MSL complex and a nucleosome array substrate. Reaction time (t) ranged from 2 to 180 minutes. In the '0' lane reaction the 4-MSL was omitted. Detection and quantification of H4 acetylation as in (D).

## Supplementary Figure 2

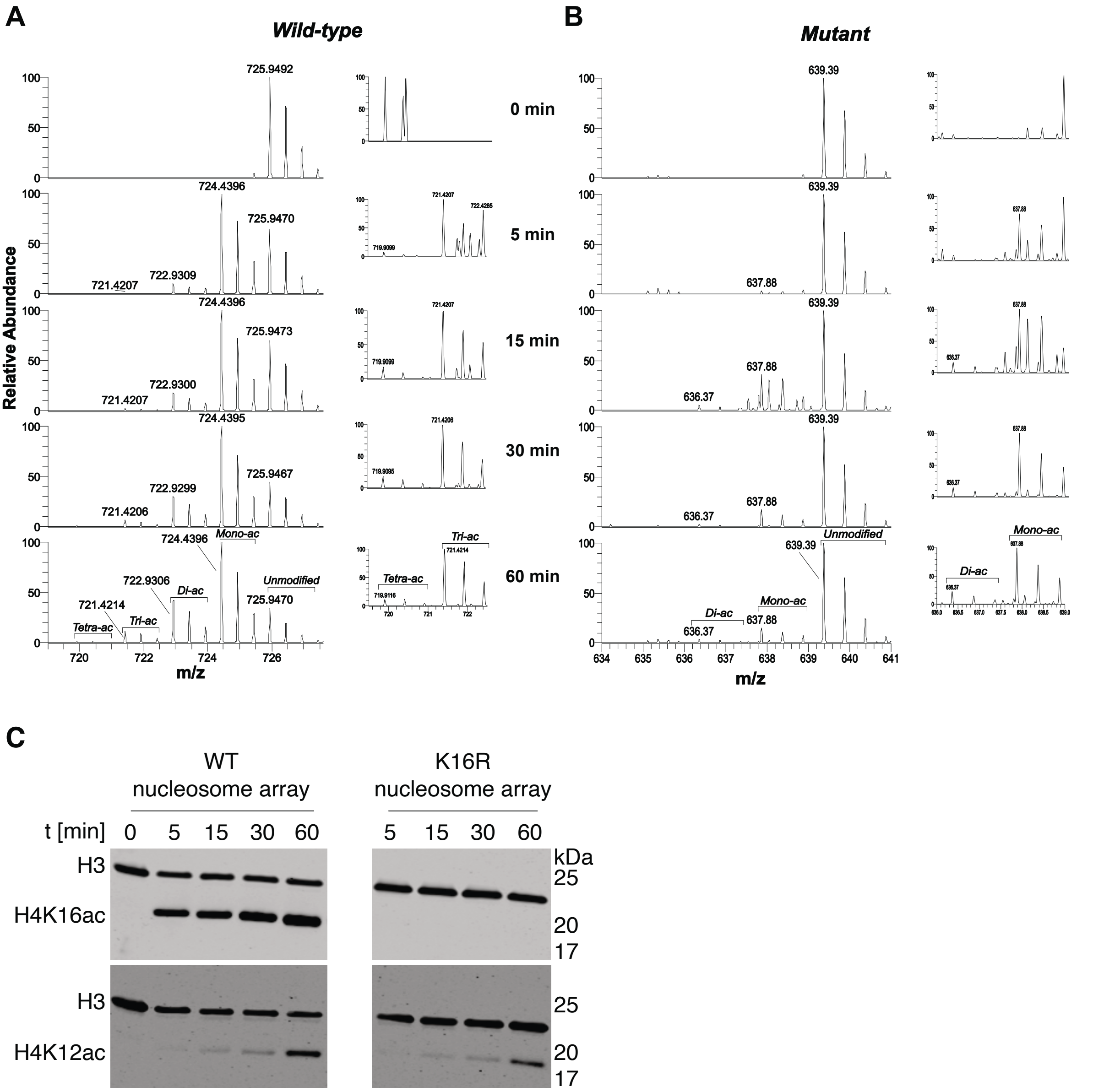


Supplementary Figure S2: The 4-MSL complex progressively acetylates H4 tail lysines *in vitro*.

(A) MS1 spectra depicting changes in the relative abundance of mono-acetylation (Mono-ac), di-acetylation (Di-ac), tri-acetylation (Tri-ac) and tetra-acetylation (Tetra-ac) in Histone 4 (H4) for wild-type at different time points. m/z values are shown only for the precursor ions. Data corresponding to Figure 2 (replicate 3 shown).

(B) MS1 spectra depicting the time-dependent changes in the relative abundance of mono-acetylation (Mono-ac) and di-acetylation (Di-ac) for the H4K16R mutant. Tri-acetylation spectra were not distinctly observed. Data corresponding to Figure 4 (replicate 3 shown).

(C) Histone acetylation assay with 50 nM 4-MSL complex on wild type or H4K16R mutant nucleosome arrays, respectively. H4 acetylation at indicated reaction times was analyzed by Western blot using antibodies specific for H4K16ac and H4K12ac. One representative Western blot is shown.

## Supplementary Figure 3

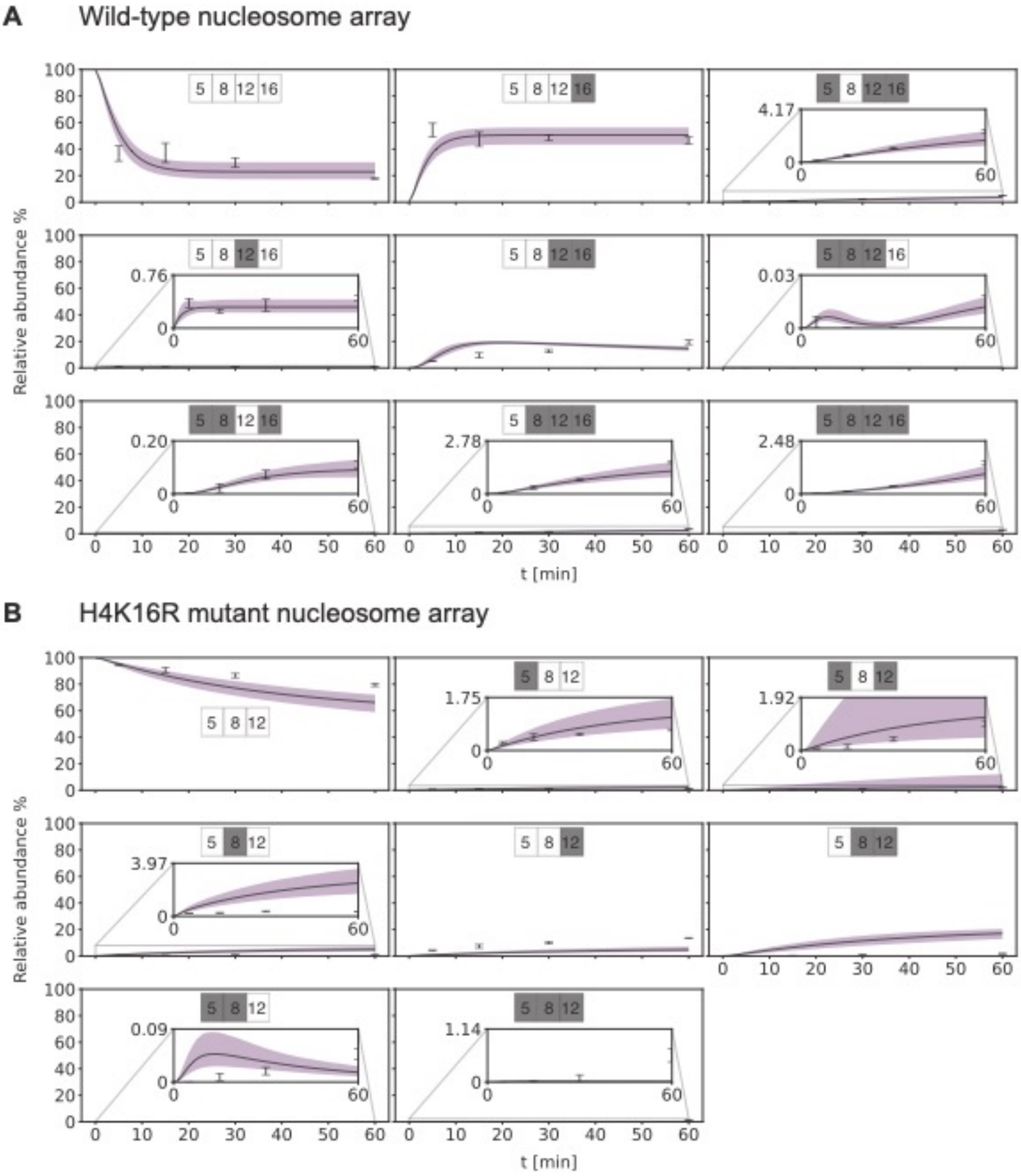


Supplementary Figure S3: H4 tail acetylation kinetics by MOF is described by a processive model.

(A) Ensemble simulation of the model fit based on data obtained with wild-type nucleosome arrays (Figure 2). An ensemble of MCMC samples were simulated. Solid black lines are the median time course trajectory. Shaded regions indicate the 99% credibility interval. Vertical bars indicate the mass spectrometry measurements as mean and standard error of the mean (SEM).

(B) Ensemble simulation of the model prediction based on data obtained with H4K16R mutant nucleosome arrays (Figure 4). The ensemble of MCMC samples used for (A) were adjusted to represent the mutant H4K16R by disabling all reactions involving K16. Solid black lines are the median time course trajectory. Shaded regions indicate the 99% credibility interval. Vertical bars indicate the mass spectrometry measurements as mean and standard error of the mean (SEM).

## Supplementary Figure 4

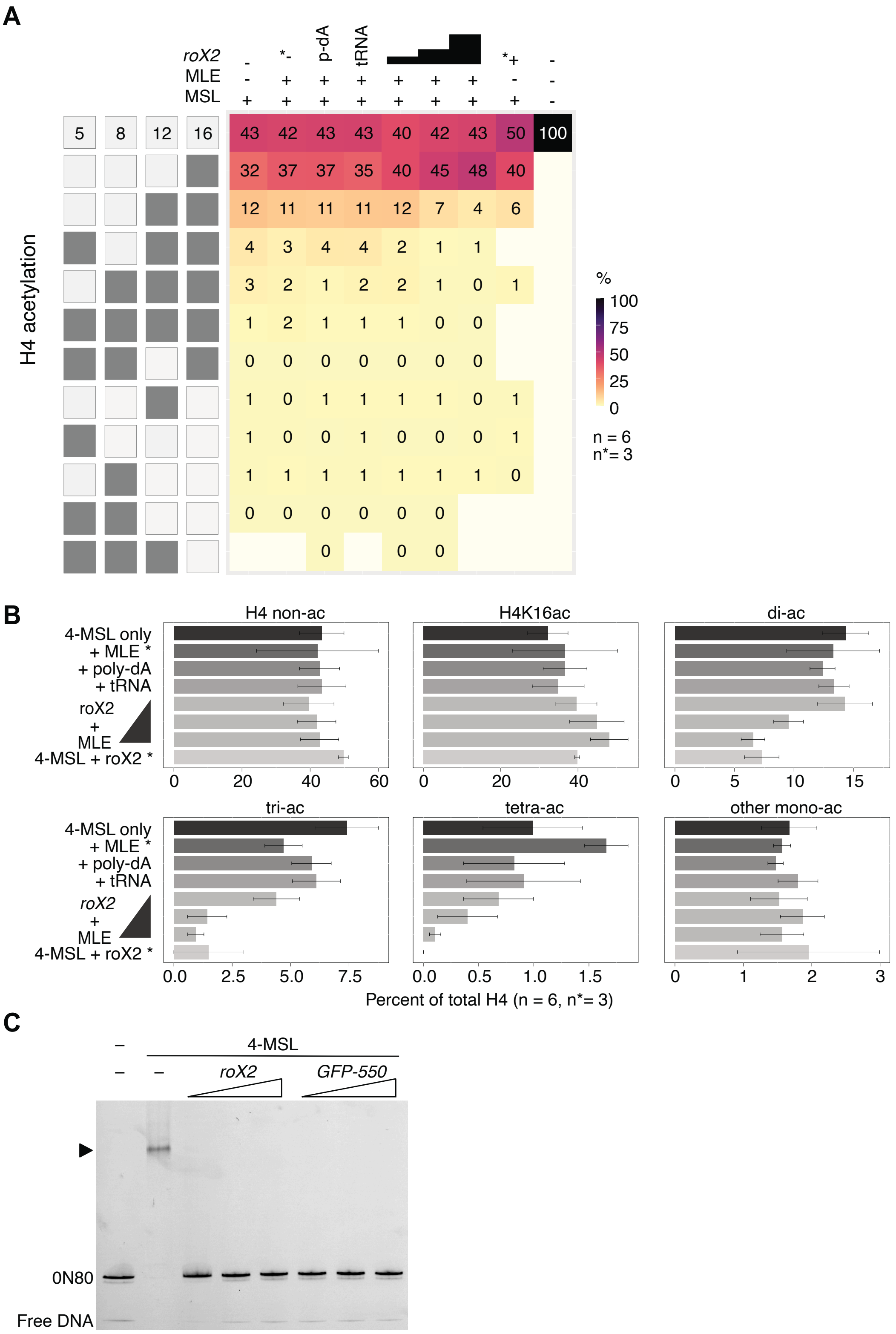


Supplementary Figure S4: RNA suppresses H4 oligo-acetylation by the 4-MSL complex *in vitro*.

(A) Heatmap displaying the abundance of individual H4 acetylation patterns in absence or presence of nucleic acids as quantified by mass spectrometry. Histone acetylation assay on nucleosome arrays was performed with 50 nM 4-MSL complex in absence or presence of 50 nM MLE, *roX2* RNA, poly-deoxyadenylic acid (p-dA) and tRNA, respectively. *roX2* RNA was

added in a 0.5-fold, 1-fold or 2-fold molar ratio to the 4-MSL complex. P-dA and tRNA were added in a 2-fold mass ratio excess. All reactions contained ATP. Reaction time was 60 min. The left panel displays combinatorial acetylation motifs on H4 lysines 5, 8, 12 and 16. Light shading displays non-acetylated residues, while dark shading means acetylation at corresponding position. The map shows all combinations that can be measured by MS2 and thus lacks some diacetylated forms. First row shows the mean levels of non-acetylated H4 at different conditions. Data represent the mean of six replicates or, when labeled with an asterisk, of three replicates.

(B) Bar plot summarizing the abundance of mono- and oligo-acetylated H4 tail motifs detected in (A). 'di-/tri-ac' represents the sum of all possible di- or tri-acetylated H4 tail motifs. 'tetra-ac' refers to the fully acetylated H4 tail. 'other mono-ac' cumulates levels of K5ac, K8ac and K12ac. Standard error of the mean is given.

(C) RNA suppresses binding to mononucleosomes by the 4-MSL complex. Complex formation of 50 nM 4-MSL and 10 nM 0N80 mononucleosomes monitored by electrophoretic mobility shift assay (EMSA). Complexes are destabilized in presence of full-length roX2 (552 bases) and GFP (550 bases) RNA, respectively, at a concentration of 25-50-100 nM each.

## Supplementary Figure 5

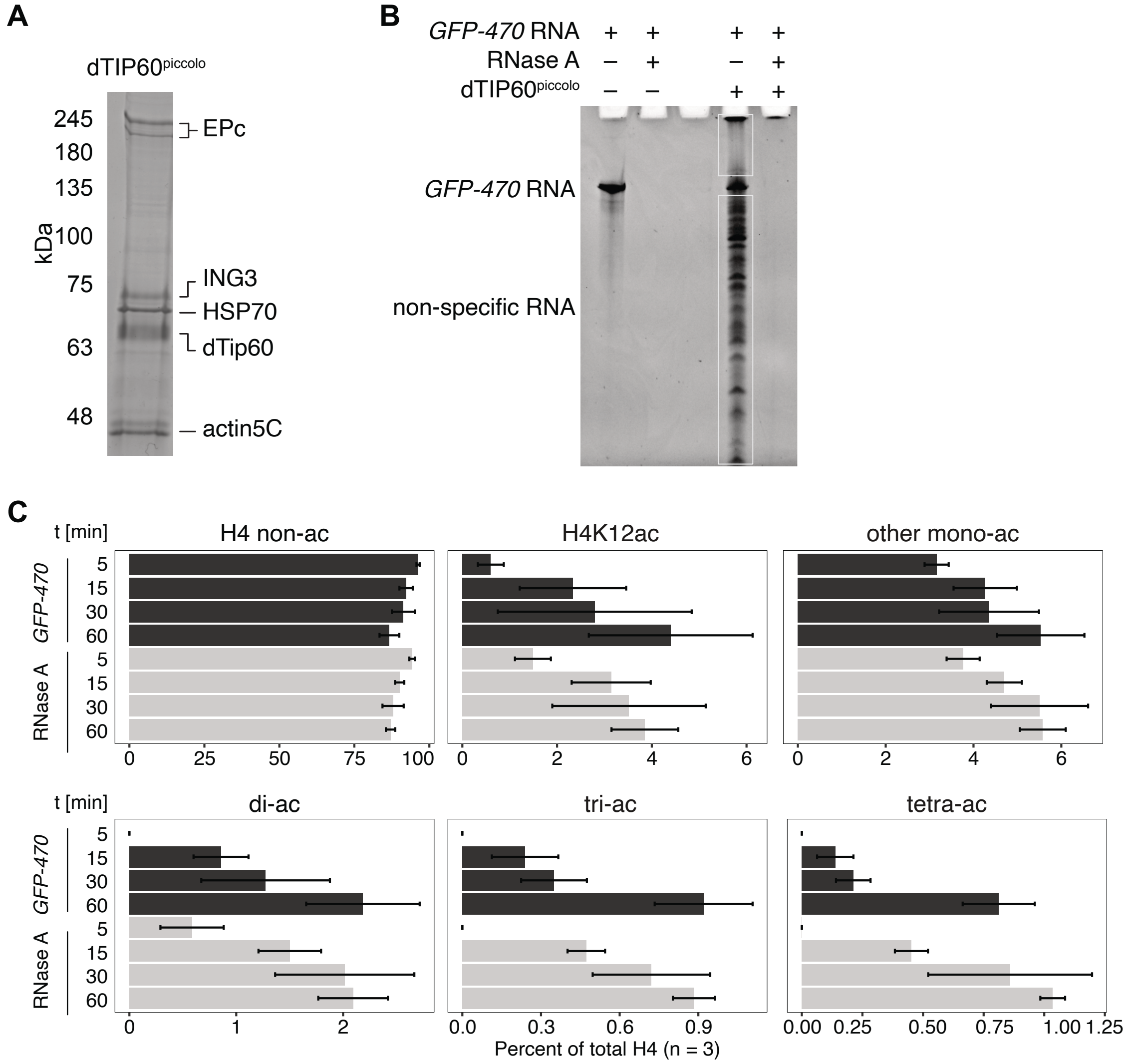


Supplementary Figure S5: The histone acetyltransferase activity of dTip60piccolo is not influenced by RNA

(A) Coomassie-stained SDS-PAGE showing purified recombinant *Drosophila melanogaster* dTip60piccolo complex composed of E(Pc), ING3 and Tip60. The 3 subunits, co-purified Sf21 actin5C and contaminating HSP70 were identified by mass spectrometry.

(B) Denaturing PAGE showing the RNA included (or digested) in the experiment shown in Figure 7. The dTip60piccolo is heavily contaminated by Sf21 RNA (white boxes). When indicated, 100 nM *GFP-470* RNA and 0.2 µg RNase A were added to the sample.

(C) Bar plot showing the abundance of H4 tail peptides acetylated by 50 nM dTip60piccolo on nucleosome arrays at indicated time points. Reactions were performed in presence of a 2-fold molar excess *GFP-470* RNA and RNase A, respectively. 'di-/tri-ac' represents the sum of all possible di- or tri-acetylated H4 tail motifs. 'tetra-ac' refers to the fully acetylated H4 tail. 'other mono-ac' cumulates levels of K5ac, K8ac and K16ac. Standard error of the mean of 3 independent protein preparations.

**Supplementary Table:**

Sequences of oligonucleotides used in this study

| Primer | Sequence (5'-3') | Purpose |
|---|---|---|
| dH4_K16R fw | GGGTGGCGCCcgtCGTCATCGCA | Cloning of the H4K16R mutant |
| dH4_K16R rv | TTTCCCAAGCCTTTGCCTC | Cloning of the H4K16R mutant |
| pACEBac1_F | TCTAGAGCCTGCAGTCTCG | Cloning of dTip60$^{piccolo}$ |
| pACEBac1_R | ATATTTATAGGTTTTTTTATTACAAAACTG | Cloning of dTip60$^{piccolo}$ |
| TwinStrep-Tip60_F | taataaaaaaacctataaatatGAGCGCATGGAGTCATCC | Cloning of dTip60$^{piccolo}$ |
| TwinStrep-Tip60_R | tcgagactgcaggctctagaTCATTTGGAGCGCTTGGAC | Cloning of dTip60$^{piccolo}$ |
| Ing3_PH_f | tcccggtccgaagcgcgcggaattcATGCTTTACCTCGAAGAC | Cloning of dTip60$^{piccolo}$ |
| Ing3_PH_r | tcctctagtacttctcgacaagcttTCAGTTCTTTCGGTTGCC | Cloning of dTip60$^{piccolo}$ |
| E(Pc)_PH_f | tcccggtccgaagcgcgcggaattcATGTCCAAGCTGTCGTTC | Cloning of dTip60$^{piccolo}$ |
| E(Pc)_PH_r | tcctctagtacttctcgacaagcttTCATCTGTTGATGGTTGAC | Cloning of dTip60$^{piccolo}$ |
| 0N80.fw | CCTGGAGAATCCCGGTGCCGAG | PCR amplification of 0N80 mononucleosomal DNA |
| 0N80_6FAM.rv | GGTACCCGGGGATCCTCTAGA | PCR amplification of 0N80 mononucleosomal DNA |
| GFP-fw76ntRNA | CACGCCGTAGGTCAGGGTGGTC | PCR primer GFP-76 template for in vitro transcription |

| | | |
|---|---|---|
| GFP-fw182ntRNA | CGTCGTCCTTGAAGAAGATGGTGCGCTC | PCR primer GFP-182 template for in vitro transcription |
| GFP-fw283ntRNA | CAGGATGTTGCCGTCCTCCTTGAAGTCGA | PCR primer GFP-283 template for in vitro transcription |
| GFP-fw391ntRNA | GTCCTCGATGTTGTGGCGGATCTTGAAGTTCA | PCR primer GFP-391 template for in vitro transcription |
| GFP-rv470ntRNA | CCTGAAGTTCATCTGCACCA | PCR primer GFP-470 template for in vitro transcription, in combination with fw GFP-RNAi primer |
| GFP-fw550ntRNA | GAGCTGGACGGCGACGTAAAC | PCR primer GFP-550 template for in vitro transcription |
| GFP-fw627ntRNA | CCACCGGTCGCCACCATG | PCR primer GFP-627 template for in vitro transcription |
| rvGFP-RNAi-primer with T7 promotor | TTAATACGACTCACTATAGGGCCTGAAGTTCATCTGCACCA | PCR primer GFP templates for in vitro transcription |
| fwGFP-RNAi | TTAATACGACTCACTATAGGGTGCTGAGGTAGTGGTTGTCG | PCR primer GFP-470 template for in vitro transcription, in combination with 470nt rv primer |

Supplementary Note 1:

Calculations to derive motif abundance from MS quantifier for K16R mutated histone H4 motifs

$$\text{H4\_noPTM\_R} = \frac{\text{MS1\_G4R16\_noPTM\_K16R\_precursor}}{\mathit{sum}(\text{MS1\_G4R16\_1Ac\_K16R\_precursor,MS1\_G4R16\_2Ac\_K16R\_precursor,} \\ \text{MS1\_G4R16\_3Ac\_K16R\_precursor,MS1\_G4R16\_noPTM\_K16R\_precursor})}$$

$$\text{H4K12ac\_R} = \frac{\text{MS1\_G4R16\_1Ac\_K16R\_precursor}}{\mathit{sum}(\text{MS1\_G4R16\_1Ac\_K16R\_precursor,MS1\_G4R16\_2Ac\_K16R\_precursor,} \\ \text{MS1\_G4R16\_3Ac\_K16R\_precursor,MS1\_G4R16\_noPTM\_K16R\_precursor})} * \frac{\text{MS2\_G4R16\_1Ac\_y8\_K12ac\_757\_K16R\_y8}}{\mathit{sum}(\text{MS2\_G4R16\_1Ac\_y8\_K12ac\_757\_K16R\_y8,MS2\_G4R16\_1Ac\_y8\_K12NoAc\_760\_K16R\_y8})}$$

$$\text{H4K8ac\_R} = \frac{\text{MS1\_G4R16\_1Ac\_K16R\_precursor}}{\mathit{sumsum}(\text{MS1\_G4R16\_1Ac\_K16R\_precursor,MS1\_G4R16\_2Ac\_K16R\_precursor,} \\ \text{MS1\_G4R16\_3Ac\_K16R\_precursor,MS1\_G4R16\_noPTM\_K16R\_precursor})} * \frac{\text{MS2\_G4R16\_y10\_1Ac\_K8K12.1Ac\_987\_K16R\_y10}}{\mathit{sumsum}(\text{MS2\_G4R16\_1Ac\_y10\_K8K12.0Ac\_990\_K16R\_y10,MS2\_G4R16\_y10\_1Ac\_K8K12.1Ac\_987\_K16R\_y10})} - \frac{\text{MS2\_G4R16\_1Ac\_y8\_K12ac\_757\_K16R\_y8}}{\mathit{sum}(\text{MS2\_G4R16\_1Ac\_y8\_K12ac\_757\_K16R\_y8,MS2\_G4R16\_1Ac\_y8\_K12NoAc\_760\_K16R\_y8})}$$

$$\text{H4K5ac\_R} = (\text{H4K12ac\_R} + \text{H4K8ac\_R}) - \frac{\text{MS1\_G4R16\_1Ac\_K16R\_precursor}}{\mathit{sumsum}(\text{MS1\_G4R16\_1Ac\_K16R\_precursor,MS1\_G4R16\_2Ac\_K16R\_precursor,} \\ \text{MS1\_G4R16\_3Ac\_K16R\_precursor,MS1\_G4R16\_noPTM\_K16R\_precursor})}$$

$$\text{H4K12K8ac\_R} = \frac{\text{MS1\_G4R16\_2Ac\_K16R\_precursor}}{\mathit{sum}(\text{MS1\_G4R16\_1Ac\_K16R\_precursor,MS1\_G4R16\_2Ac\_K16R\_precursor,} \\ \text{MS1\_G4R16\_3Ac\_K16R\_precursor,MS1\_G4R16\_noPTM\_K16R\_precursor})} * \frac{\text{MS2\_G4R16\_2Ac\_b3\_K5NoAc\_288\_K16R\_b3}}{\mathit{sum}(\text{MS2\_G4R16\_2Ac\_b3\_K5Ac\_285\_K16R\_b3,MS2\_G4R16\_2Ac\_b3\_K5NoAc\_288\_K16R\_b3})} \text{ <-}$$

$$\text{H4K5K8ac\_R} = \frac{\text{MS1\_G4R16\_2Ac\_K16R\_precursor}}{\mathit{sum}(\text{MS1\_G4R16\_1Ac\_K16R\_precursor,MS1\_G4R16\_2Ac\_K16R\_precursor,} \\ \text{MS1\_G4R16\_3Ac\_K16R\_precursor,MS1\_G4R16\_noPTM\_K16R\_precursor})} * \frac{\text{MS2\_G4R16\_2Ac\_y8\_K12NoAc\_760\_K16R\_y8}}{\mathit{sum}(\text{MS2\_G4R16\_2Ac\_y8\_K12Ac\_757\_K16R\_y8,MS2\_G4R16\_2Ac\_y8\_K12NoAc\_760\_K16R\_y8})}$$

$$\text{H4K5K12ac\_R} = (\text{H4K12K8ac\_R} + \text{H4K5K8ac\_R}) - \frac{\text{MS1\_G4R16\_2Ac\_K16R\_precursor}}{\mathit{sum}(\text{MS1\_G4R16\_1Ac\_K16R\_precursor,MS1\_G4R16\_2Ac\_K16R\_precursor,} \\ \text{MS1\_G4R16\_3Ac\_K16R\_precursor,MS1\_G4R16\_noPTM\_K16R\_precursor})}$$

$$\text{H4K5K8K12ac\_R} = \frac{\text{MS1\_G4R16\_3Ac\_K16R\_precursor}}{\mathit{sum}(\text{MS1\_G4R16\_1Ac\_K16R\_precursor,MS1\_G4R16\_2Ac\_K16R\_precursor,} \\ \text{MS1\_G4R16\_3Ac\_K16R\_precursor,MS1\_G4R16\_noPTM\_K16R\_precursor})}$$

# Supplementary Note 2

## Data availability

All scripts, data, and models used to produce the mathematical modeling results are available at [4].

## Ordinary differential equation models

As H4 has 4 acetylation sites (K5, K8, K12, and K16), there exist $2^4 = 16$ motifs (acetylation states). Each motif can be acetylated at an unacetylated site.

We used ordinary differential equation models (ODEs) to describe the processes associated with enzymatic acetylation of histone H4. These ODEs represent the instantaneous change in the abundances of H4 motifs. Solving the ODEs over time can provides timecourse simulations or predictions for H4 motif abundances.

Consider some H4 motif M , all reactions that can acetylate another motif to produce M (input reactions $I_M$ ), and all reactions that can acetylate M into another motif (output reactions $O_M$ ).

Then, given the simplest reaction kinetic, mass action, the ODE for M is

$$\frac{dM}{dt} = \sum_{i \in I_M} k_i M_i - \sum_{o \in O_M} k_o M, \tag{1}$$

where t is time, k are reaction rate constants, and $M_i$ are the corresponding motifs for those reactions.

Under the Michaelis-Menten kinetic assumption, the ODE for M is

$$\frac{dM}{dt} = \sum_{i \in I_M} \frac{k_{cat,i} E_0 M_i}{K_{m,i} + M_i} - \sum_{o \in O_M} \frac{k_{cat,o} E_0 M}{K_{m,o} + M}, \tag{2}$$

where $k_{cat}$ are the catalysis rate constants, $K_m$ are the Michaelis constants (equal to $\frac{k_r + k_{cat}}{k_f}$, given the notation in Figure 3A), and $E_0$ is the total amount of 4-MSL in the system.,

With the processive kinetic assumption, $M$ is now described by the system of ODEs

$$\frac{dM}{dt} = -k_f M E + k_r M_E \tag{3}$$

$$\frac{dM_E}{dt} = \sum_{i \in I_M} k_{cat,i} M_{E,i} + k_f M E - k_r M_E - \sum_{o \in O_M} k_{cat,o} M_E . \tag{4}$$

(5)

where $M_E$ is the enzyme-bound $M$. In the processive case, the enzyme abundance is directly modeled by the ODE

$$\frac{dE}{dt} = \sum_{i \in M} k_{r,i} M_{E,i} - k_{f,i} M_i E, \tag{6}$$

where $M$ is an index over all motifs.

## Parameter estimation

The ODEs in the previous section can be written in the general form

$$\frac{dx}{dt} = f(x(t,\theta,u),\theta,u), \quad x(t_0,\theta,u) = x_0(\theta,u), \tag{7}$$

in which $x(t, \theta, u) \in \mathbb{R}^{nx}_{+}$ is the vector of state variables, $\theta \in \mathbb{R}^{n\theta}_{+}$ is the parameter vector, $u \in \mathbb{R}^{nu}_{+}$ is the vector of constant inputs, $t \in \mathbb{R}$ is the time, and the initial condition at the initial timepoint $t_0$ is $x_0 : \mathbb{R}^{n\theta}_{+} \times \mathbb{R}^{nu}_{+} \to \mathbb{R}^{nx}_{+}$.

In our case, where the ODEs are generated from the enzyme kinetics described in the previous section, then x is the abundances of motifs and enzyme, θ is all parameters *k*, and u is experiment-specific data, e.g. the amount of enzyme used.

The connection between data and the model is provided by the output map $h : \mathbb{R}^{nx}_{+} \times \mathbb{R}^{n\theta}_{+} \times \mathbb{R}^{nu}_{+} \to \mathbb{R}^{ny}$ and the output vector,

$$y(t,\theta,u) = h(x(t,\theta,u),\theta,u). \tag{8}$$

We estimate θ with by maximizing the likelihood *L*, which is the probability of the measurements given the model [5]. In the case of independent, Gaussian-distributed measurements, the objective function based on the negative log-likelihood is given by

$$J(\theta) = -\log L(\theta) = \frac{1}{2}\sum_{i=1}^{ny}\sum_{j=1}^{nt}\left(\log\left(2\pi\sigma_{ij}^2\right) + \left(\frac{\bar{y}_{ij} - y_i(t_j,\theta,u)}{s_{ij}}\right)^2\right), \tag{9}$$

where $\bar{y}_{ij} = y_i(t_j,\theta,u) + \varepsilon_{ij}$ are measurements with additive noise $\varepsilon_{ij} \sim N(0, s_{ij}^2)$, $s$ are standard deviations, and $y_i(t_j,\theta)$ are model simulations. Here, we define $s_{ij} = \sigma_{ij}\, y_i(t_j,\theta,u)$, where $\sigma_{ij}$ is estimated, for scale-dependent noise. The maximum likelihood estimate is the solution to the optimization problem

$$\theta^* = \arg\min_\theta J(\theta).$$

To solve this estimation problem, we used gradient-based optimization, as provided by the combination of the tools AMICI [2], pyPESTO [3], and Fides [1]. AMICI provides objective function evaluations and sensitivity-based gradients, which are used by Fides to find $\theta^*$, with pyPESTO as the interface between AMICI and Fides. Multi-start optimization was performed with 1000 starts, with the BFGS Hessian update scheme in Fides.
Computations were performed on the Unicorn cluster (University of Bonn, Germany) with 1 compute node, which has 1 TB RAM, 1 TD SSD, and either 2x AMD EPYC 7F72 3.20 GHz or 2x AMD EPYC 7443 2.85 GHz CPUs.

## Uncertainty analysis

To incorporate model uncertainty into our predictions, we performed Markov chain Monte Carlo (MCMC) sampling, using the adaptive Metropolis-Hastings algorithm with the adaptive parallel tempering (10 chains) algorithm, as implemented in pyPESTO [3]. 100,000 samples were generated, and the converged samples (by Geweke's test) were used to create an ensemble of parameter vectors, from which predictions were made. 99% credibility intervals were generated by simulating the ensemble of samples, then discarding the 1% most extreme (farthest from the median) simulated values at each time point. Computations were done on 2 CPU cores of a compute node on the Unicorn cluster.

## References

[1] Fabian Fröhlich and Peter K. Sorger. Fides: Reliable Trust-Region Optimization for Parameter Estimation of Ordinary Differential Equation Models. Feb. 2022. doi: 10.1101/2021.05.20.445065.

[2] Fabian Fröhlich et al. “AMICI: High-Performance Sensitivity Analysis for Large Ordinary Differential Equation Models”. In: Bioinformatics 37.20 (Oct. 2021), pp. 3676–3677. issn: 1367-4803. doi: 10.1093/bioinformatics/btab227.

[3] Yannik Schälte et al. pyPESTO: A Modular and Scalable Tool for Parameter Estimation for Dynamic Models. May 2023. doi: 10.48550/arXiv.2305.01821. arXiv: 2305.01821 [q-bio, stat].

[4] “Supplementary Scripts for Kiss et. al (2023)”. doi: 10.5281/zenodo.10221453.

[5] Alejandro F Villaverde et al. “A Protocol for Dynamic Model Calibration”. In: Briefings in Bioinformatics 23.1 (Jan. 2022), bbab387. issn: 1477-4054. doi: 10.1093/bib/bbab387.

## Supplementary Note 3: R script for quantification of relative H4 acetylation levels

```
# Load the required packages


library(tcltk)
library(readxl)
library(ggpubr)
library(ggsignif)
library(data.table)
library(purrr)
library(reshape2)
library(ggplot2)
library(ggforce)
library(gdata)

####################################
# Get the current working directory
getwd()

# Choose the directory if the current working directory is not the one required
setwd(tclvalue(tkchooseDirectory()))

# Choose the output file from SkyLine
data_file <- tk_choose.files()
# The output file usually contains the following columns:
# Peptide Note,
# Peptide,
# Precursor,
# Protein,
# Replicate,
# Peptide Peak Found Ratio,
# Peptide Retention Time,
# Transistion Result,
# Fragment Ion,
# Total Area MS1

############################

# Load the SkyLine output file
tab_data <- read.csv(data_file, header = TRUE)

# Remove unnecessary columns
tab_data <- tab_data[, -c(2,3,6,7)]
```

```
# Change column names and remove unwanted data

# Change the column names as per requirement
colnames(tab_data) <- c("Peptide_Name",
                        "Protein",
                        "Sample_Name",
                        "Peak_Area",
                        "Fragment_ion",
                        "Total_Area_MS1")

# Removing any QC samples
tab_data <- tab_data[!grepl("QC", tab_data$Sample_Name),]
# Removing any blank samples
tab_data <- tab_data[!grepl("blank", tab_data$Sample_Name),]
# Removing those peptides that were not identified/quantified properly
tab_data <- tab_data[!grepl("DO_NOT_USE", tab_data$Peptide_Name),]

# Reset the row names
rownames(tab_data) <- c()

# Combine information in Peptide name and Fragment ion and remove Peptide Name
# and Fragment ion column
tab_data$Peptide_Fragment_Name <- paste(tab_data$Peptide_Name, tab_data$Fragment_ion, sep="_")
tab_data <- tab_data[, -c(1,5)]

# Change the column order
tab_data <- tab_data[,c(5,1,2,3,4)]

# Arrange the data either in increase/decreasing order of either the sample or values
tab_data <-tab_data[order(tab_data$Sample_Name, decreasing = FALSE),]

# Reset the row names
rownames(tab_data) <- c()

# Remove hidden \n from the Peptide_Name column
tab_data <- as.data.frame(sapply(tab_data, function(x) { gsub("[\r\n]", "", x) }))

# Convert Peak Area to numeric
tab_data$Peak_Area <- as.character(tab_data$Peak_Area)
tab_data$Peak_Area <- as.numeric(tab_data$Peak_Area)

# Convert Total MS1 area to numeric
tab_data$Total_Area_MS1 <- as.character(tab_data$Total_Area_MS1)
tab_data$Total_Area_MS1 <- as.numeric(tab_data$Total_Area_MS1)

# Get all the sample names
Samples <- unique(tab_data$Sample_Name)

#############################

# Create an object of class "List" for all of the possible H4 modifications in wildtype
H4_noPTM <- list()
H4K5ac <- list()
```

```
H4K8ac <- list()
H4K12ac <- list()
H4K16ac <- list()
H4K5K8ac <- list()
H4K12K16ac <- list()
H4K5K8K12ac <- list()
H4K5K8K16ac <- list()
H4K5K12K16ac <- list()
H4K8K12K16ac <- list()
H4K5K8K12K16ac <- list()

# Create an object of class "List" for all of the possible H4 modifications in mutant
H4_noPTM_R <- list()
H4K5ac_R <- list()
H4K8ac_R <- list()
H4K12ac_R <- list()
H4K12K8ac_R <- list()
H4K5K8ac_R <- list()
H4K5K12ac_R <- list()
H4K5K8K12ac_R <-list()

###############################

# For loop to calculate the relative ratios of each modifications in wild-type and
# mutant H4 (check Feller et al., 2015 and supplementary file for corresponding formulas)

for (i in 1:length(Samples)){
  temp <- tab_data[tab_data$Sample_Name==Samples[i],] # Obtain the data
  # based on Sample names

  # Make the first column as rownames
  rownames(temp) <- temp[,1]
  temp[,1] <- NULL

  ## List of H4 modifications. Calculations for motifs at H4 are
  # provided in Feller C, et al., 2015

  H4_noPTM[i] <- temp["MS1_G4R17_noPTM_precursor",4]/
    sum(temp["MS1_G4R17_1Ac_precursor",4],
        temp["MS1_G4R17_2Ac_precursor",4],
        temp["MS1_G4R17_3Ac_precursor",4],
        temp["MS1_G4R17_4Ac_precursor",4],
        temp["MS1_G4R17_noPTM_precursor",4])

  H4K5ac[i] <- (temp["MS1_G4R17_1Ac_precursor",4]/
                  sum(temp["MS1_G4R17_1Ac_precursor",4],
                      temp["MS1_G4R17_2Ac_precursor",4],
                      temp["MS1_G4R17_3Ac_precursor",4],
                      temp["MS1_G4R17_4Ac_precursor",4],
                      temp["MS1_G4R17_noPTM_precursor",4])) *
    (1 - (temp["MS2_G4R17_1Ac_y12_K16K12K8.1Ac_1217_y12",3]/
            sum(temp["MS2_G4R17_1Ac_y12_K16K12K8.1Ac_1217_y12",3],
                temp["MS2_G4R17_1Ac_y12_K16K12K8.0Ac_1220_y12",3])))
```

```
H4K8ac[i] <- (temp["MS1_G4R17_1Ac_precursor",4]/
                    sum(temp["MS1_G4R17_1Ac_precursor",4],
                        temp["MS1_G4R17_2Ac_precursor",4],
                        temp["MS1_G4R17_3Ac_precursor",4],
                        temp["MS1_G4R17_4Ac_precursor",4],
                        temp["MS1_G4R17_noPTM_precursor",4])) *
  (temp["MS2_G4R17_1Ac_y12_K16K12K8.1Ac_1217_y12",3]/
     sum(temp["MS2_G4R17_1Ac_y12_K16K12K8.1Ac_1217_y12",3],
         temp["MS2_G4R17_1Ac_y12_K16K12K8.0Ac_1220_y12",3]) -
     temp["MS2_G4R17_1Ac_y7_K16K12.1Ac_760_y7",3]/
     sum(temp["MS2_G4R17_1Ac_y7_K16K12.1Ac_760_y7",3],
         temp["MS2_G4R17_1Ac_y7_K16K12.0Ac_763_y7",3]))

H4K12ac[i] <- (temp["MS1_G4R17_1Ac_precursor",4]/
                    sum(temp["MS1_G4R17_1Ac_precursor",4],
                        temp["MS1_G4R17_2Ac_precursor",4],
                        temp["MS1_G4R17_3Ac_precursor",4],
                        temp["MS1_G4R17_4Ac_precursor",4],
                        temp["MS1_G4R17_noPTM_precursor",4])) *
  ((temp["MS2_G4R17_1Ac_y7_K16K12.1Ac_760_y7",3]/
      sum(temp["MS2_G4R17_1Ac_y7_K16K12.1Ac_760_y7",3],
          temp["MS2_G4R17_1Ac_y7_K16K12.0Ac_763_y7",3])) -
     (temp["MS2_G4R17_1Ac_y5_K16ac_530_y5",3]/
        sum(temp["MS2_G4R17_1Ac_y5_K16ac_530_y5",3],
            temp["MS2_G4R17_1Ac_y5_K16NoAc_533_y5",3])))

H4K16ac[i] <- (temp["MS1_G4R17_1Ac_precursor",4]/
                    sum(temp["MS1_G4R17_1Ac_precursor",4],
                        temp["MS1_G4R17_2Ac_precursor",4],
                        temp["MS1_G4R17_3Ac_precursor",4],
                        temp["MS1_G4R17_4Ac_precursor",4],
                        temp["MS1_G4R17_noPTM_precursor",4])) *
  (temp["MS2_G4R17_1Ac_y5_K16ac_530_y5",3]/
     sum(temp["MS2_G4R17_1Ac_y5_K16ac_530_y5",3],
         temp["MS2_G4R17_1Ac_y5_K16NoAc_533_y5",3]))

H4K5K8ac[i] <- (temp["MS1_G4R17_2Ac_precursor",4]/
                    sum(temp["MS1_G4R17_1Ac_precursor",4],
                        temp["MS1_G4R17_2Ac_precursor",4],
                        temp["MS1_G4R17_3Ac_precursor",4],
                        temp["MS1_G4R17_4Ac_precursor",4],
                        temp["MS1_G4R17_noPTM_precursor",4])) *
  (temp["MS2_G4R17_2Ac_y7_K16K12.0Ac_763_y7",3]/
     sum(temp["MS2_G4R17_2Ac_y7_K16K12.2Ac_757_y7",3],
         temp["MS2_G4R17_2Ac_y7_K16K12.1Ac_760_y7",3],
         temp["MS2_G4R17_2Ac_y7_K16K12.0Ac_763_y7",3]))

H4K12K16ac[i] <- (temp["MS1_G4R17_2Ac_precursor",4]/
                    sum(temp["MS1_G4R17_1Ac_precursor",4],
                        temp["MS1_G4R17_2Ac_precursor",4],
                        temp["MS1_G4R17_3Ac_precursor",4],
                        temp["MS1_G4R17_4Ac_precursor",4],
                        temp["MS1_G4R17_noPTM_precursor",4])) *
```

```
    (temp["MS2_G4R17_2Ac_y7_K16K12.2Ac_757_y7",3]/
       sum(temp["MS2_G4R17_2Ac_y7_K16K12.0Ac_763_y7",3],
           temp["MS2_G4R17_2Ac_y7_K16K12.1Ac_760_y7",3],
           temp["MS2_G4R17_2Ac_y7_K16K12.2Ac_757_y7",3]))

  H4K5K8K12ac[i] <- (temp["MS1_G4R17_3Ac_precursor",4]/
                                     sum(temp["MS1_G4R17_1Ac_precursor",4],
                                         temp["MS1_G4R17_2Ac_precursor",4],
                                         temp["MS1_G4R17_3Ac_precursor",4],
                                         temp["MS1_G4R17_4Ac_precursor",4],
                                         temp["MS1_G4R17_noPTM_precursor",4])) *
    (1-(temp["MS2_G4R17_3Ac_y5_K16ac_530_y5",3]/
          sum(temp["MS2_G4R17_3Ac_y5_K16ac_530_y5",3],
              temp["MS2_G4R17_3Ac_y5_K16NoAc_533_y5",3])))

  H4K5K8K16ac[i] <- (temp["MS1_G4R17_3Ac_precursor",4]/
                                     sum(temp["MS1_G4R17_1Ac_precursor",4],
                                         temp["MS1_G4R17_2Ac_precursor",4],
                                         temp["MS1_G4R17_3Ac_precursor",4],
                                         temp["MS1_G4R17_4Ac_precursor",4],
                                         temp["MS1_G4R17_noPTM_precursor",4])) *
    ((temp["MS2_G4R17_3Ac_y5_K16ac_530_y5",3]/
        sum(temp["MS2_G4R17_3Ac_y5_K16ac_530_y5",3],
            temp["MS2_G4R17_3Ac_y5_K16NoAc_533_y5",3]))-
       (temp["MS2_G4R17_3Ac_y7_K16K12.2Ac_757_y7",3]/
          sum(temp["MS2_G4R17_3Ac_y7_K16K12.2Ac_757_y7",3],
              temp["MS2_G4R17_3Ac_y7_K16K12.1Ac_760_y7",3])))

  H4K5K12K16ac[i] <- (temp["MS1_G4R17_3Ac_precursor",4]/
                                      sum(temp["MS1_G4R17_1Ac_precursor",4],
                                          temp["MS1_G4R17_2Ac_precursor",4],
                                          temp["MS1_G4R17_3Ac_precursor",4],
                                          temp["MS1_G4R17_4Ac_precursor",4],
                                          temp["MS1_G4R17_noPTM_precursor",4])) *
    ((temp["MS2_G4R17_3Ac_y7_K16K12.2Ac_757_y7",3]/
        sum(temp["MS2_G4R17_3Ac_y7_K16K12.2Ac_757_y7",3],
            temp["MS2_G4R17_3Ac_y7_K16K12.1Ac_760_y7",3]))-
       (temp["MS2_G4R17_3Ac_y12_K16K12K8.3Ac_1211_y12",3]/
          sum(temp["MS2_G4R17_3Ac_y12_K16K12K8.3Ac_1211_y12",3],
              temp["MS2_G4R17_3Ac_y12_K16K12K8.2Ac_1214_y12",3])))

  H4K8K12K16ac[i] <- (temp["MS1_G4R17_3Ac_precursor",4]/
                                      sum(temp["MS1_G4R17_1Ac_precursor",4],
                                          temp["MS1_G4R17_2Ac_precursor",4],
                                          temp["MS1_G4R17_3Ac_precursor",4],
                                          temp["MS1_G4R17_4Ac_precursor",4],
                                          temp["MS1_G4R17_noPTM_precursor",4])) *
    (temp["MS2_G4R17_3Ac_y12_K16K12K8.3Ac_1211_y12",3]/
       sum(temp["MS2_G4R17_3Ac_y12_K16K12K8.3Ac_1211_y12",3],
           temp["MS2_G4R17_3Ac_y12_K16K12K8.2Ac_1214_y12",3]))

  H4K5K8K12K16ac[i] <- temp["MS1_G4R17_4Ac_precursor",4]/
    sum(temp["MS1_G4R17_noPTM_precursor",4],
```

```
          temp["MS1_G4R17_1Ac_precursor",4],
          temp["MS1_G4R17_2Ac_precursor",4],
          temp["MS1_G4R17_3Ac_precursor",4],
          temp["MS1_G4R17_4Ac_precursor",4])

## List of H4 modifications in mutant
# Calculations for motifs are given in the supplementary

H4_noPTM_R[i] <- temp["MS1_G4R16_noPTM_K16R_precursor",4]/
   sum(temp["MS1_G4R16_1Ac_K16R_precursor",4],
          temp["MS1_G4R16_2Ac_K16R_precursor",4],
          temp["MS1_G4R16_3Ac_K16R_precursor",4],
          temp["MS1_G4R16_noPTM_K16R_precursor",4])

H4K12ac_R[i] <- (temp["MS1_G4R16_1Ac_K16R_precursor",4]/
                                  sum(temp["MS1_G4R16_1Ac_K16R_precursor",4],
                                         temp["MS1_G4R16_2Ac_K16R_precursor",4],
                                         temp["MS1_G4R16_3Ac_K16R_precursor",4],
                                         temp["MS1_G4R16_noPTM_K16R_precursor",4])) *
   (temp["MS2_G4R16_1Ac_y8_K12ac_757_K16R_y8",3]/
       sum(temp["MS2_G4R16_1Ac_y8_K12ac_757_K16R_y8", 3],
             temp["MS2_G4R16_1Ac_y8_K12NoAc_760_K16R_y8",3]))

H4K8ac_R[i] <- (temp["MS1_G4R16_1Ac_K16R_precursor",4]/
                                 sum(temp["MS1_G4R16_1Ac_K16R_precursor",4],
                                        temp["MS1_G4R16_2Ac_K16R_precursor",4],
                                        temp["MS1_G4R16_3Ac_K16R_precursor",4],
                                        temp["MS1_G4R16_noPTM_K16R_precursor",4])) *
   ((temp["MS2_G4R16_y10_1Ac_K8K12.1Ac_987_K16R_y10",3]/
          sum(temp["MS2_G4R16_1Ac_y10_K8K12.0Ac_990_K16R_y10", 3],
                temp["MS2_G4R16_y10_1Ac_K8K12.1Ac_987_K16R_y10",3])) -
       (temp["MS2_G4R16_1Ac_y8_K12ac_757_K16R_y8",3]/
            sum(temp["MS2_G4R16_1Ac_y8_K12ac_757_K16R_y8", 3],
                  temp["MS2_G4R16_1Ac_y8_K12NoAc_760_K16R_y8",3])))

H4K5ac_R[i] <-     (H4K12ac_R[[i]] + H4K8ac_R[[i]]) -
   (temp["MS1_G4R16_1Ac_K16R_precursor",4]/
       sum(temp["MS1_G4R16_1Ac_K16R_precursor",4],
             temp["MS1_G4R16_2Ac_K16R_precursor",4],
             temp["MS1_G4R16_3Ac_K16R_precursor",4],
             temp["MS1_G4R16_noPTM_K16R_precursor",4]))

H4K12K8ac_R[i] <- (temp["MS1_G4R16_2Ac_K16R_precursor",4]/
                                    sum(temp["MS1_G4R16_1Ac_K16R_precursor",4],
                                           temp["MS1_G4R16_2Ac_K16R_precursor",4],
                                           temp["MS1_G4R16_3Ac_K16R_precursor",4],
                                           temp["MS1_G4R16_noPTM_K16R_precursor",4])) *
   (temp["MS2_G4R16_2Ac_b3_K5NoAc_288_K16R_b3", 3]/
       sum(temp["MS2_G4R16_2Ac_b3_K5Ac_285_K16R_b3", 3],
             temp["MS2_G4R16_2Ac_b3_K5NoAc_288_K16R_b3", 3]))

H4K5K8ac_R[i] <- (temp["MS1_G4R16_2Ac_K16R_precursor",4]/
                                   sum(temp["MS1_G4R16_1Ac_K16R_precursor",4],
```

```
                                       temp["MS1_G4R16_2Ac_K16R_precursor",4],
                                       temp["MS1_G4R16_3Ac_K16R_precursor",4],
                                       temp["MS1_G4R16_noPTM_K16R_precursor",4])) *
      (temp["MS2_G4R16_2Ac_y8_K12NoAc_760_K16R_y8", 3]/
          sum(temp["MS2_G4R16_2Ac_y8_K12Ac_757_K16R_y8", 3],
                temp["MS2_G4R16_2Ac_y8_K12NoAc_760_K16R_y8", 3]))

   H4K5K12ac_R[i] <- (H4K12K8ac_R[[i]] + H4K5K8ac_R[[i]]) -
      temp["MS1_G4R16_2Ac_K16R_precursor",4]/
      sum(temp["MS1_G4R16_1Ac_K16R_precursor",4],
            temp["MS1_G4R16_2Ac_K16R_precursor",4],
            temp["MS1_G4R16_3Ac_K16R_precursor",4],
            temp["MS1_G4R16_noPTM_K16R_precursor",4])

   H4K5K8K12ac_R[i] <- temp["MS1_G4R16_3Ac_K16R_precursor",4]/
      sum(temp["MS1_G4R16_1Ac_K16R_precursor",4], temp["MS1_G4R16_2Ac_K16R_precursor",4],
            temp["MS1_G4R16_3Ac_K16R_precursor",4], temp["MS1_G4R16_noPTM_K16R_precursor",4])
   }

##################################################

# Rename the list with corresponding sample names

# H4 modifications in wild-type
names(H4_noPTM) <- Samples
names(H4K5ac) <- Samples
names(H4K8ac) <- Samples
names(H4K12ac) <- Samples
names(H4K16ac) <- Samples
names(H4K5K8ac) <- Samples
names(H4K12K16ac) <- Samples
names(H4K5K8K12ac) <- Samples
names(H4K5K8K16ac) <- Samples
names(H4K5K12K16ac) <- Samples
names(H4K8K12K16ac) <- Samples
names(H4K5K8K12K16ac) <- Samples

# H4 modifications in mutant
names(H4_noPTM_R) <- Samples
names(H4K5ac_R) <- Samples
names(H4K8ac_R) <- Samples
names(H4K12ac_R) <- Samples
names(H4K12K8ac_R) <- Samples
names(H4K5K8ac_R) <- Samples
names(H4K5K12ac_R) <- Samples
names(H4K5K8K12ac_R) <-Samples

###########################################
# Convert all the lists in the environment to nested list
myList <- Filter(function(x) is(x, "list"), mget(ls()))

# Convert the list with names to dataframe
result.df <- map_df(myList, ~data.frame("Sample" = .x), .id="Histone Modifications")
```

```
# Remove word "Sample" to match it with corresponding ID in the SampleTable
colnames(result.df) <- gsub("Sample.", "", colnames(result.df))

# Load the sample table that has information about the sample and corresponding ID
SampleTable <- readxl::read_xlsx(tk_choose.files(), sheet = 1, col_names = TRUE)

#   Check if the order and name of the sample ID is the same in the loaded file and
# the data frame
stopifnot(identical(as.character(SampleTable$Sample_ID),
                    gsub("Sample.","", colnames(result.df)[2:ncol(result.df)])))

# Replace sample ID with Sample Name
colnames(result.df)[2:ncol(result.df)] <- SampleTable$Sample_Name

# Multiple by 100 to convert into percentages
result <- abs(result.df[,-1]) * 100

# Change the rownames to corresponding histone modification
rownames(result) <- result.df[,1]

# Write the output
write.csv(x = result, file = tclvalue(tkgetSaveFile()))

###########################


# knitr::opts_chunk$set(echo = TRUE)
```
